# Dynamic interplay of *WRKY*, *GRAS*, and *ERF* transcription factor families in tomato-endophytic fungal symbiosis: insights from transcriptome and genome-wide analysis

**DOI:** 10.3389/fpls.2023.1181227

**Published:** 2023-06-05

**Authors:** Ibrahim Khan, Lubna, Sajjad Asaf, Rahmatullah Jan, Saqib Bilal, Abdul Latif Khan, Kyung-Min Kim, Ahmed Al-Harrasi

**Affiliations:** ^1^Natural and Medical Sciences Research Center, University of Nizwa, Nizwa, Oman; ^2^Department of Applied Biosciences, Kyungpook National University, Daegu, Republic of Korea; ^3^Department of Engineering Technology, University of Houston, Sugar Land, TX, United States

**Keywords:** *S. lycopersicum*, *SlWRKY*, phylogenetic analysis, symbiotic association, *C. lunata*

## Abstract

Plant-microbe interactions play a crucial role in shaping plant growth and development, as well as in mediating plant responses to biotic and abiotic stresses. In this study, we used RNA-seq data to examine the expression profiles of *SlWRKY*, *SlGRAS*, and *SlERF* genes during the symbiotic association of *Curvularia lunata* SL1 with tomato (*Solanum lycopersicum*) plants. We also conducted functional annotation analysis by comparative genomics studies of their paralogs and orthologs genes, as well as other approaches, such as gene analysis and protein interaction networks, to identify and characterize the regulatory roles of these TFs in the development of the symbiotic association. We found that more than half of the investigated *SlWRKY* genes exhibited significant upregulation during symbiotic association, including *SlWRKY*38, *SlWRKY*46, *SlWRKY*19, and *SlWRKY*51. Several *SlGRAS* and *SlERF* genes were upregulated, such as *SlGLD*2, *SlGLD*1, *SlERF.C.5*, *ERF16*, and *SlERF.B12*. Conversely, a smaller proportion of *SlWRKY*, *SlGRAS*, and *SlERF* genes were significantly downregulated during symbiotic association. Furthermore, we investigated the possible roles of *SlWRKY*, *SlGRAS*, and *SlERF* genes in hormonal regulation during plant-microbe interactions. We identified several upregulated candidate transcripts likely to be involved in plant hormone signaling pathways. Our findings are consistent with previous studies on these genes, providing further evidence of their involvement in hormonal regulation during plant-microbe interactions. To validate the RNA-seq data accuracy, we performed RT-qPCR analyses of selected *SlWRKY*, *SlGRAS*, and *SlERF* genes, which showed similar expression patterns to those observed in the RNA-seq data. These results confirmed the accuracy of our RNA-seq data and provided additional support for the differential expression of these genes during plant-microbe interactions. Taken together, our study provides new insights into the differential expression profiles of *SlWRKY*, *SlGRAS*, and *SlERF* genes during symbiotic association with *C. lunata*, as well as their potential roles in hormonal regulation during plant-microbe interactions. These findings could be useful for guiding future research on the ways in which plants and microbes interact, and may ultimately lead to the creation of better approaches for promoting plant growth under stressful conditions.

## Introduction

Endophytic fungi are an endosymbiotic group of microorganisms that colonize the healthy internal tissues of living plants and cause no apparent symptoms of disease in the host plants (Dutta et al., 2014). The plant provides photosynthetic sugars to the fungal symbiont, and the fungus supplies available mineral nutrients such as phosphorus and nitrogen in the soil (Daguerre et al., 2017; Lubna et al., 2018). This association is beneficial to plants because it either promotes growth directly by producing plant growth-promoting substances or indirectly by inhibiting the growth of phytopathogens (Kapulnik and Okon, 2002). Approximately 2 million endophytic fungal species have been identified, forming mutualistic relationships with more than 20,000 different plant species (Hawksworth, 2001). Endophytic fungi have been recognized as an important and novel resource of natural bioactive compounds with potential applications in various fields, including agriculture, medicine, and the food industry (Zhao et al., 2010). It has been reported that the symbiotic association of fungi with plants promotes nutrient uptake and production of phytohormones, resulting in increased plant growth and yield without supplementing extensive fertilizers (Kurepin et al., 2014). The constant discovery of endophytic fungi in different plant species and their potential to produce a wide range of extracellular hydrolytic enzymes, alkaloids, and other physiologically active chemical compounds have sparked intense interest among scientists worldwide (Abdel-Azeem et al., 2019; Castro et al., 2022). Endophytes promote plant growth by secreting phytohormones such as gibberellins (GAs) and indole acetic acid (IAA) which consequently aid in improving nutrient uptake through bidirectional nutrient transfer and improving plant health by protecting them from phytopathogens (Lubna et al., 2018; Andreozzi et al., 2019). Plant-endophytic fungal association contributes to plant protection against harmful environmental conditions such as increased heavy metals, drought, and salinity by triggering tolerance (de Carvalho et al., 2020). Several studies have shown that endophytes enhance plant growth and resistance to different biotic and abiotic stresses; therefore, we can believe that plant growth promotion, when triggered by endophytes, will indirectly support the host plant defense mechanisms against adverse environmental conditions (Kuldau and Bacon, 2008; Fadiji and Babalola, 2020; González-Teuber et al., 2022; Lubna et al., 2022; Bilal et al., 2023; Zheng et al., 2023). The occurrence of extreme conditions such as pathogenicity, drought, salinity, and heavy metals dramatically enhance ethylene secretion, which may result in alteration of the cellular processes and reduced growth and, ultimately, plant death. However, some endophytes have reported producing 1-aminocyclopropane-1-carboxylate (ACC) deaminase, which cleaves the ethylene precursor, ACC, into ammonia and 2-oxobutanoate to decrease ethylene levels in the plants (Bhattacharyya and Jha, 2012; Nascimento et al., 2014). Induced systematic resistance (ISR) and systematic acquired resistance (SAR) are the two induced resistance mechanisms that are moderated by phytohormones, including ethylene, jasmonic acid, and salicylic acid where plant defenses have been preconditioned by prior infection or treatment that results in resistance against subsequent challenge by a pathogen or parasite (Fadiji and Babalola, 2020; Kamle et al., 2020). Previously it was reported that some endophytes like *Penicillium thiomii* produce antioxidants that significantly prevent diseases caused by oxygen-derived free radicals and reactive oxygen species (ROS) (Cui et al., 2015). Altogether, symbiosis development results in substantial and coordinated transcriptional reprogramming in both partners (Martin et al., 2016). However, the regulatory mechanisms triggering and controlling the expression of fungal and plant signaling genes and the developmental pathways leading to endophytic symbiosis are largely unknown.

Transcription factors (TFs) are essential DNA-binding proteins in regulating gene expression. For correct gene regulation in response to various developmental and environmental signals, all organisms depend on TFs, the trans elements of the gene expression system, to interact with cis-regulatory (CRRs) of DNA (Khan et al., 2022). The two major TFs involved in upregulating and down-regulating gene expression in a very controlled manner are activators and repressors, respectively. (Todeschini et al., 2014; Khan et al., 2021). Activators generally exhibit one or more sequence-specific DNA-binding domains (DBD) and one activation domain (AD). DBD recognizes and binds to specific DNA sequences in the promoter region to regulate gene expression, while the ADs recruit and interact with basal transcriptional machinery to mediate transcription initiation (Titz et al., 2006; Lin et al., 2010). In *S. lycopersicum*, more than 1800 TFs are identified, which are classified into 58 different families based on conserved motifs and unique structure of DNA-binding domains (DBDs) (Tian et al., 2020). Previously, various genome and transcriptome-wise studies have been employed to investigate the tomato (*S. lycopersicum*) TFs at high resolution and depth in different contexts. However, no comprehensive reports exist to identify and characterize the TFs involved in *S. lycopersicum*-fungus symbiosis. The *WRKY* TFs are one of the largest families of TFs and are called jack of all trades because they regulate various developmental and adaptation processes in plants such as seed dormancy and germination, plant growth and development, hormones response pathways, morphogenesis of trichomes, senescence, biosynthesis of secondary metabolites and various biotic and abiotic stresses (Huang et al., 2012; Phukan et al., 2016; Jiang et al., 2017; Wang et al., 2017b; Yao et al., 2022). *GRAS* proteins reportedly play a crucial regulatory role in various developmental and physiological processes, such as induced resistance to elevated levels of intracellular reactive oxygen species (ROS) (Czikkel and Maxwell, 2007), controlling shoot development (Chen et al., 2019), meristem development, and signal transduction (Sun et al., 2012) and facilitate symbiosis-specific association (Pimprikar et al., 2016). Ethylene-responsive factors (ERF) are a plant-specific family that significantly regulates plant growth, development, and responses to biotic and abiotic stresses (Cui et al., 2021; Yang et al., 2021). Recently it is reported that members of the *ERF* family are involved in conferring resistance to fungal invasion by regulating signaling pathways of salicylic acid (SA), jasmonic acid (JA), and ethylene (ET) (Yang et al., 2021). The detailed composition and mode of action of *WRKY*, *GRAS*, and *ERF* TFs are well explored (Huang et al., 2012; Yang et al., 2021; Waseem et al., 2022). Here we will focus on the comparative genomic and transcriptomic studies and potential functional roles of these TFs in tomato plants, particularly during *S. lycopersicum*- *C. lunata* interaction.

In the current study, we identified and functionally characterized the *SlWRKY*, *SlGRAS*, and *SlERF* TF families in the *S. lycopersicum* genome by performing various in silico analyses such as phylogenetic relationships, gene structure, conserved motifs, gene ontology, protein-protein interaction, co-expression patterns, and gene expression profiling. In this study, we also utilized RNA-seq data to investigate the regulatory roles of these TF families during the *S. lycopersicum* and *C. lunata* symbiotic association.

## Materials and methods

### Sequences retrieval

We retrieved the genes encoding the *WRKY*, *GRAS*, and *ERF* TFs of *S. lycopersicum* and *Arabidopsis* from the Plant Transcription Factor Database v5.0 (http://planttfdb.gao-lab.org/, accessed on 6 December 2022). The corresponding genome sequences and protein sequences were downloaded from Solanaceae Genomics Network (SGN, https://solgenomics.net/, accessed on 6 December 2022), Phytozome 13 (https://phytozome-next.jgi.doe.gov/, accessed on 6 December 2022), and Arabidopsis Information Resource (TAIR, https://www.arabidopsis.org/index.jsp, accessed on 6 December 2022). Consequently, 81 *SlWRKY*, 72 *AtWRKY*, 54 *SlGRAS*, 37 *AtGRAS*, 137 *SlERF*, and 139 *AtERF* genes were identified. For valid identification and confirming the presence of the respective domains of the obtained protein sequences, the Simple Modular Architecture Research Tool (SMART, http://smart.embl.de/, accessed on 6 December 2022) was used at E-value <10^-5^. This data was used for subsequent analysis.

### Phylogenetic reconstruction and classification

Multiple sequence alignment and phylogenetic analysis were employed to classify the *SlWRKY, SlGRAS*, and *SlERF* TFs in their respective phylogenetic groups. First, the deduced protein sequences were aligned using the ClustalW progressive alignment method. Parameters for gap opening penalty and gap extension penalty in pairwise and multiple sequence alignment were set at 15.00 and 6.66, respectively. The phylogenetic tree construction was performed by the neighbor-joining method of the MEGA 11 software, with 1000 times bootstrap replicates (BS) (Tamura et al., 2021). In order to study the phylogenetic and paralogous relationships of *SlWRKY*, *SlGRAS*, and *SlERF* proteins, individual phylogenetic trees were constructed while for determining their orthologous relationships with their Arabidopsis counterparts, combined phylogenetic trees were constructed using the MEGA 11 software. The resulting trees were visualized and annotated using FigTree software v 1.4.4 (https://mybiosoftware.com/figtree-1-3-1-produce-figures-phylogenetic-trees.html).

### Chromosomal location and gene duplication

Solanaceae Genomics Network (SGN, https://solgenomics.net/, accessed on 6 December 2022) supports us by the chromosomal location information of *S. lycopersicum WRKY*, *GRAS*, and *ERF* genes. The MapInspect software (https://mapinspect.software.informer.com/, accessed on 6 December 2022) was utilized to map the genes on chromosomes by putting the starting and ending positions with respective accession numbers. The plant genome duplication database (PGDD, http://chibba.agtec.uga.edu/duplication/, accessed on 6 December 2022) was used to execute the duplicate chromosomal blocks and then identify the *WRKY*, *GRAS*, and *ERF* genes in the duplication block, which allowed us to identify duplicate *WRKY*, *GRAS* and *ERF* genes of *S.lycopersicum* (Lee et al., 2012). The TFs with ≥ 70% similar aligned sequences of the entire gene length were defined as duplicated genes. Genes separated by five or fewer gene loci within a physical distance of 100 kb were considered tandem duplicates [41], and those co-paralogs located on duplicated chromosomal blocks were considered segmental duplicates (Wei et al., 2007).

### Gene exon-intron organization and motif analysis

The exon and intron organization of individual *WRKY*, *GRAS*, and *ERF* genes were illustrated by the online tools of the Gene Structure Display Server program (GSDS 2.0, http://gsds.gao-lab.org/, accessed on 7 December 2022)(Hu et al., 2015) by aligning the genomic sequences with the CDS sequences. In order to identify the potentially conserved motif in *WRKY*, *GRAS*, and *ERF* TFs, deduced amino acids sequences were analyzed using the online server the Multiple EM for Motif Elicitation (MEME 5.4.1, https://meme-suite.org/meme/doc/meme.html, accessed on 7 December 2022)(Bailey et al., 2006), with the following parameters: number of repetitions, any; maximum number of motifs, 15; and optimum width set to ≥ 6 and ≤ 200 amino acids residues.

### Gene ontology enrichment analysis

GO enrichment analysis was performed by agriGO gene ontology enrichment analysis tool (agriGO, http://bioinfo.cau.edu.cn/agriGO/, accessed on 7 December 2022) with the TopGO ‘elim’ algorithm for the three hierarchies’: biological process, molecular functions, and cellular components. The loci of *WRKY*, *GRAS*, and *ERF* genes were used as queries and the *S. lycopersicum* Genome database (PLantGDB, http://plantgdb.org/SlGDB/, accessed on 7 December 2022) was used for the singular enrichment analysis. Hypergeometric distribution with a P-value cutoff of 0.05 and a term-mapping count cutoff of 5 were used to calculate the GO term enrichment. Obtained P-values from Fisher’s exact test were adjusted with the FDR for multiple comparisons to detect over-represented GO terms. GO terms with both P-values and FDR < 0.05 were considered significantly enriched.

### Prediction of protein-protein interaction network

All the putative protein sequences of *WRKY*, *GRAS*, and *ERF* in FASTA format were submitted to the online server STRING version 11.5 (https://string-db.org, accessed on 8 December 2022), with the organism specified as *S. lycopersicum* to predict the protein-protein interaction networks and functional annotations. After the blast step was finished, genes with significantly high confidence scores were used to construct the network. Genes that did not interact with any others were removed.

### Growth conditions, fungal inoculation, and RNA Extraction

In the current study, tomato seeds (*S. lycopersicum* cv. Yegwang) was used at the experimental greenhouse of Kyungpook National University, South Korea. First, the seeds were surface sterilized with 10% hypochlorous acid and 70% ethanol and washed with autoclaved distilled water to remove the impurities. The soaked seeds have been germinated on hygiene filter paper in an incubator at 30°C in dark conditions. After successful sprouting, the seeds were planted in plastic pots in a greenhouse. The greenhouse temperature was maintained at 28 ± 2°C, 50 ± 5% relative humidity, and 16-hr-light/8-hr-dark photoperiod. The experiment was set up as follows: (a) control plants (distilled water), (b) plants inoculated with endophytic fungus strain *C. lunata* SL1, which were all grown in a growth chamber and subjected to a 24-h cycle at 25°C for 10 h and 28°C for 14 h. After every three days, the plants were inoculated with the fungal culture under the same growth conditions. Four replicates were prepared per treatment. Leaf samples were collected randomly, immediately placed in liquid nitrogen, and stored at -80°C freezer until analysis.

### Expression pattern analysis *WRKY*, *GRAS*, and *ERF* genes during fungal symbiotic association with tomato plants

RNA was isolated from fresh leaves of *S. lycopersicum* plants after the SL1 fungal inoculation, with purification and library construction. The total RNA of each sample was diluted to 100 ng and used for cDNA libraries preparation. Libraries from three different replicates of each treatment were sequenced and analyzed. The Illumina HiSeq2000 sequencing platform was utilized for RNA-seq analysis according to manufacturer’s prescribed procedure [43], which resulted in 51-bp single-end reads. An efficient computational pipeline was used to find differences in gene regulation between the inoculated and non-inoculated plants. The pipeline included the following steps: (1) using FastQC (Andrews, 2010) for the quality check; (2) using Trim Galore (Krueger, 2012) for data trimming; (3) HISAT2 (Sirén et al., 2014) for indexing and alignment to reference genome;(4) read count quantification using Feature Count (subread_v2.0.2) and (5) using DESeq2 (Love et al., 2014) in R program for differential gene expression analysis.

### Reverse transcription-quantitative PCR

qRT-PCR was performed to authenticate the results of the RNA-seq dataset of the selected genes. The first standard cDNA was produced using PCR BIOSYSTEMS’s qPCRBIO cDNA Synthesis Kits after total RNA was diluted to a final concentration of 100 ng/μl. qRT-PCR was conducted using BioFACTTM 2× Real-Time PCR Master Mix (Including SYBR^®^ Green I). The PCR reaction was carried out in a 20 μl total volume containing 10 μl Master Mix, 1 μl cDNA,1 μl primer, and 6 μl RNase free water, with each reaction repeated three times. Step One Plus Real-Time PCR System PCR machine, Life Technologies Holdings Pte Ltd. (Singapore) was used. Primer3 (https://bioinfo.ut.ee/primer3-0.4.0/) program was used to design primers for each selected gene as listed in (**Table S2**). Actin (house-keeping gene) was used as internal control, which has been previously validated as a suitable internal control in similar experiments (Li et al., 2019; Ali et al., 2021; Hussain et al., 2022) to normalize gene expression and the comparative ΔΔ Ct method of qRT-PCR was utilized to calculate the expression level of the genes in control plants compared with *C. lunata* SL1 inoculated ones. To reduce the experimental error, three biological and three technical repeats were used for each sample. The following PCR conditions were used: 10 min at 94°C, followed by 35 cycles at 94°C (45 s), 65°C (45 s), and 72°C (1 min), with an extension step at 72°C (10 min). The gene amplification threshold was set at 0.1. Each sample was run three times with three different replicates.

## Results

### Genome-wide identification of the *SlWRKY*, *SlGRAS*, and *SlERF* genes

We curated 81 *SlWRKY*, 72 *AtWRKY*, 54 *SlGRAS*, 37 *AtGRAS*, 137 *SlERF*, and 139 *AtERF* non-redundant TFs derived from PlantTFDB v.5.0 and respectively confirmed their specific domain using SMART. Consensus accession numbers of the TFs were used as queries for downloading the corresponding genome sequences and protein sequences from the web-based databases, including SGN, Phtytozome 13, and TAIR. The alternative splice forms of these TFs are neglected because their specific regulatory roles are frequently poorly understood or unavailable (Lee and Roy, 2004). The protein sequences of putative *SlWRKY*, *SlGRAS* and *SlERF* members were subjected and analyzed with EXPASY PROTOPARAM (https://web.expasy.org/protparam, accessed on 8 December 2022) and SGN to find out some basic information of these TFs including, counted exons, nucleotide length (bp) and number of amino acids (aa) (**Table S1**).

### Multiple sequence alignment and phylogenetic tree analysis

**Figure 1:**
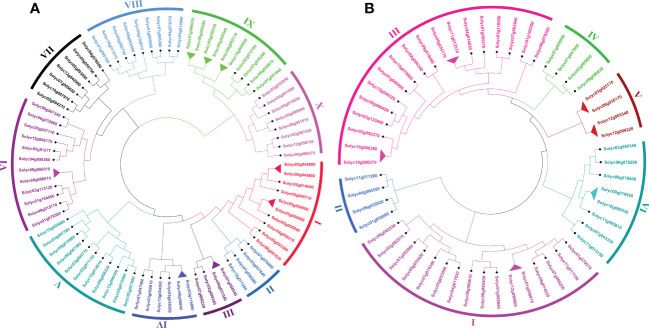
**(A)** The neighbor-joining phylogenetic tree of the 81 *SlWRKY* proteins constructed with MEGA-11 with 1000 times replicate. The major 10 phylogenetic clades are marked as I to X, respectively. **(B)** The neighbor-joining phylogenetic tree of the 54 *SlGRAS* proteins constructed with MEGA-11 with 1000 times replicate. The major 6 phylogenetic clades are marked as I to VI, respectively.

**Figure 2:**
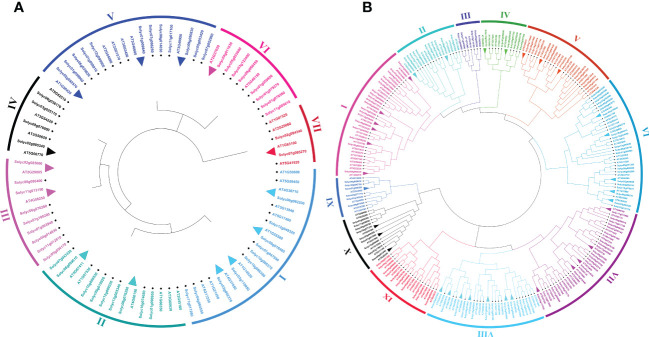
**(A)** Comparative phylogenetic tree of *GRAS* genes of Arabidopsis and *S. lycopersicum*. Multiple sequence alignment of full-length *GRAS* proteins was done using ClustalW, and the phylogenetic tree was constructed using MEGA-11 by the neighbor-joining method with 1000 bootstrap replicates. The tree was divided into 7 phylogenetic clades marked with different colors. **(B)** Comparative phylogenetic tree of *ERF* genes of Arabidopsis and *S. lycopersicum*. Multiple sequence alignment of full-length *ERF* proteins was done using ClustalW, and the phylogenetic tree was constructed using MEGA-11 by the neighbor-joining method with 1000 bootstrap replicates. The tree was divided into 11 phylogenetic clades marked with different colors.

To investigate the genome-wide phylogenetic relationship and evolutionary patterns among *WRKY*, *GRAS*, and *ERF* genes in *S. lycopersicum* and *A. thaliana*, unrooted phylogenetic trees were constructed based on the aligned full-length amino acid sequences of all the proteins. To detect the evolutionary and paralogous relationships within the *S. lycopersicum WRKY*, *GRAS*, and *ERF* TFs families, separate unrooted trees were constructed using MEGA 11 software by the neighbor-joining (NJ) method, and the inferred phylogeny was tested with 1,000 bootstraps (BS) replicates. To analyze their orthologous relationships and functional annotations, we selected the well-studied and representative plant, Arabidopsis, and combined phylogenetic trees constructed from the selected TFs families of the two plant species. As shown in **Figure 1A**, the observed sequence similarity and phylogenetic tree topology segregate *SlWRKY* TFs into 10 major clades, named clade I- clade X. The discoveries exposed that clade-VI comprised a maximum number of *SlWRKY* members (12), followed by clade-V with 11 members and clade-I and clade-IX with 10 members’ while the minimum number of *SlWRKY* members (4) were found in clade-II and clade-III. The phylogenetic tree of the *SlGRAS* TFs family grouped them into six clades, named clade-I to clade-VI. Clade-I and clade-III are larger, with 16 and 18 members of *SlGRAS* members respectively, while clade-II, IV, and V contain only 4 *SlGRAS* TFs (**Figure 1B**). Similarly, seven major clades were found in the phylogenetic tree of *SlERF* TFs. Maximum *SlERF* TFs (35) were clustered in clade-IV, followed by clade-I and clade-III with 21 and 20 members, whereas clade-VII is the smallest with 13 *SlERF* members (**Figure S1A**). We also constructed their combined phylogenetic trees to understand the evolutionary relationships among WRKY, GRAS, and ERF TFs of S. *lycopersicum* and *A. thaliana*. According to the topological tree structure and classification *WRKY*, *GRAS*, and *ERF* TFs of *S. lycopersicum* and *A. thaliana* were clustered into 9, 6, and 11 phylogenetic clades, respectively. Among the major nine clades of *WRKY* TFs, clade-I is the largest one containing 29 members, followed by clade- II with 24 members, while clade-VII is the smallest, with only 9 members (**Figure S1B**). The combined phylogenetic tree *GRAS* consists of six distinct major clades, the largest of which (clade-I) contains 20, and the smallest one (clade-IV) contains 8 members (**Figure 2A**). For *ERF* TFs, nine major clades were defined phylogenetically. Clade-VII represents the largest clade which harbored 39 members, followed by clade-I and clade-VI with 38 *ERF* members, while clade-III is the smallest clade with only 7 *ERF* members (**Figure 2B**).

### Insights from the paralogous and orthologous relationships

Paralogous and orthologous genes are two different types of homologous genes. Orthologous genes are found in different species that evolved from a common ancestor, while paralogous genes evolved by duplication events within the same genome (Koonin, 2005; Moreira and López-García, 2011). Studies showed that paralogous proteins have the same biochemical function, but their target sites may change, and orthologs are generally assumed to retain the same functions in different organisms (Mirny and Gelfand, 2002). Hence, in phylogenetics, a critical comparative genome-wide analysis of paralogous and orthologous gene pairs can be a powerful tool for the functional annotation of uncharacterized genes. The trees presented in **Figures 1A, B** and **S1A** allowed us to identify putative paralogous pairs. Careful inspection of the constructed trees revealed that 8, 6, and 26 paralogous pairs of *SlWRKY, SlGRAS*, and *SlERF* proteins are supported by strong (>50) BS scores. Comparative phylogenetic analysis *A. thaliana* and *S. lycopersicum WRKY*, *GRAS*, and *ERF* proteins revealed a total of 24, 14, and 34 orthologous pairs, respectively, with a high degree of homology (**Figure S1B**; **Figures 2A, B**).

### Homology-based prediction of gene functions

Genes that are descended from a common ancestral gene are likely to have the same functions. Therefore, homological analysis is widely used to predict the function of an uncharacterized gene (Dai et al., 2020). Since most of the *WRKY*, *GRAS*, and *ERF* TFs in *S. lycopersicum* have not been thoroughly studied in terms of their precise physiological and regulatory roles, the comparative homology-based analysis of these genes with the most extensively studied Arabidopsis counterparts will allow us to predict their specific functions. For instance, in Arabidopsis, AT4G31800 (*AtWRKY18*), the ortholog of Solyc06g068460 (*SlWRKY40*) induced expression of defense-related genes to negatively regulate AMP-triggered immunity (PTI) against *Pseudomonas syringae* bacteria and the powdery mildew fungus *Golovinomyces orontii* (Bai et al., 2018). AT2G38470 (*AtWRKY33*), ortholog of Solyc09g014990 (*SlWRKY33*), is associated with salicylic acid (SA) mediated repression of the jasmonic acid (JA) pathway, which led to induced susceptibility to *Botrytis cinerea* fungus (Birkenbihl et al., 2012). AT1G14920 (*AtGAI*), an ortholog of Solyc03g110950 of the GRAS family, negatively regulates GA signal transduction to modulate plant growth (Peng et al., 1997), and AT4G37650 (*AtSHR*), the ortholog of Solyc02g092370 play a key role in the formation of multiple layers of the cortex without an expansion of the endoderm (Wu et al., 2014). In AtERF family, AT5G51990 (*CBF4*), ortholog of Solyc01g009440 is involved to regulate adaptation to drought stress (Haake et al., 2002), and AT3G23220 (*ESE1*) and At5g25190 (*ESE3*) orthologs of Solyc09g066350 and Solyc06g065820 respectively are important salt stress regulating genes (Zhang et al., 2011). Similarly, some genes in paralogous pairs are also characterized previously. For instance, Solyc08g008280 (*SlWRKY53*) and Solyc08g082110 (*SlWRKY54*) paralog of Solyc01g095630 (*SlWRKY41*) and Solyc10g009550 (*SlWRKY42*) respectively retard the red coloration of the fruits during viral infection (Wang et al., 2017a). In *GRAS* family, the Solyc11g012510 paralog of Solyc05g054170 and Solyc12g005340 paralog of Solyc12g099220 is expressed in all the tissues indicating their crucial role in plant growth and development (Niu et al., 2017). Hence, based on these homologous relationships, we can predict the putative physiological function of their uncharacterized counterparts.

### Chromosomal distribution and gene duplication analysis

Genome chromosomal location analysis revealed that *SlGRAS* and *SlERF* genes were distributed on all chromosomes, while members of the *SlWRKY* family were not mapped to chromosome 11. The MapInspect software was used to map the genes on their corresponding chromosomes. As shown in **Figure S2**, chromosomes 5, 1, and 3 contained the maximum number of *WRKY*, *GRAS*, and *ERF* genes, respectively. Most of the genes belonging to the same phylogenetic clade were located on the same chromosome, which may be related to the homologous segments caused by polyploidy events in plant evolution (Xu et al., 2020). For instance, 80% of *WRKY* genes in clade-I of the tree were located on chromosome 5, 50% of *GRAS* genes located on chromosome 1 belong to clade-I of the tree, and about 56% *ERF* genes located on chromosome 10 belong to clade-I of the respective phylogenetic tree.

Gene duplication arising from genome-wide polyploidization, or region-specific duplication, is widely accepted as a prominent feature to contribute to the establishment of multigene families and the generation of new gene functions (Li et al., 2016; Sun et al., 2022). To reveal the potential ancestral association, we assessed intra and inter-chromosomal duplication among *SlWRKY, SlGRAS*, and *SlERF* genes. As shown in **Figure S3**, we identify approximately 20, 3, and 13 potential segmental and tandem duplicated pairs in *SlWRKY*, *SlGRAS*, and *SlERF* genes, respectively. Relatively more duplicated pairs were discovered on chromosomes 1, 2, 3, and 5, while only two gene pairs were discovered on chromosomes 4 and 9. Together, our findings indicated that most of the *SlWRKY* and *SlERF* genes arose through segmental duplication and tandem duplication, which all contributed to the expansion of these families in *S. lycopersicum*.

### Analysis of exon-intron organization and conserved motifs

**Figure 3:**
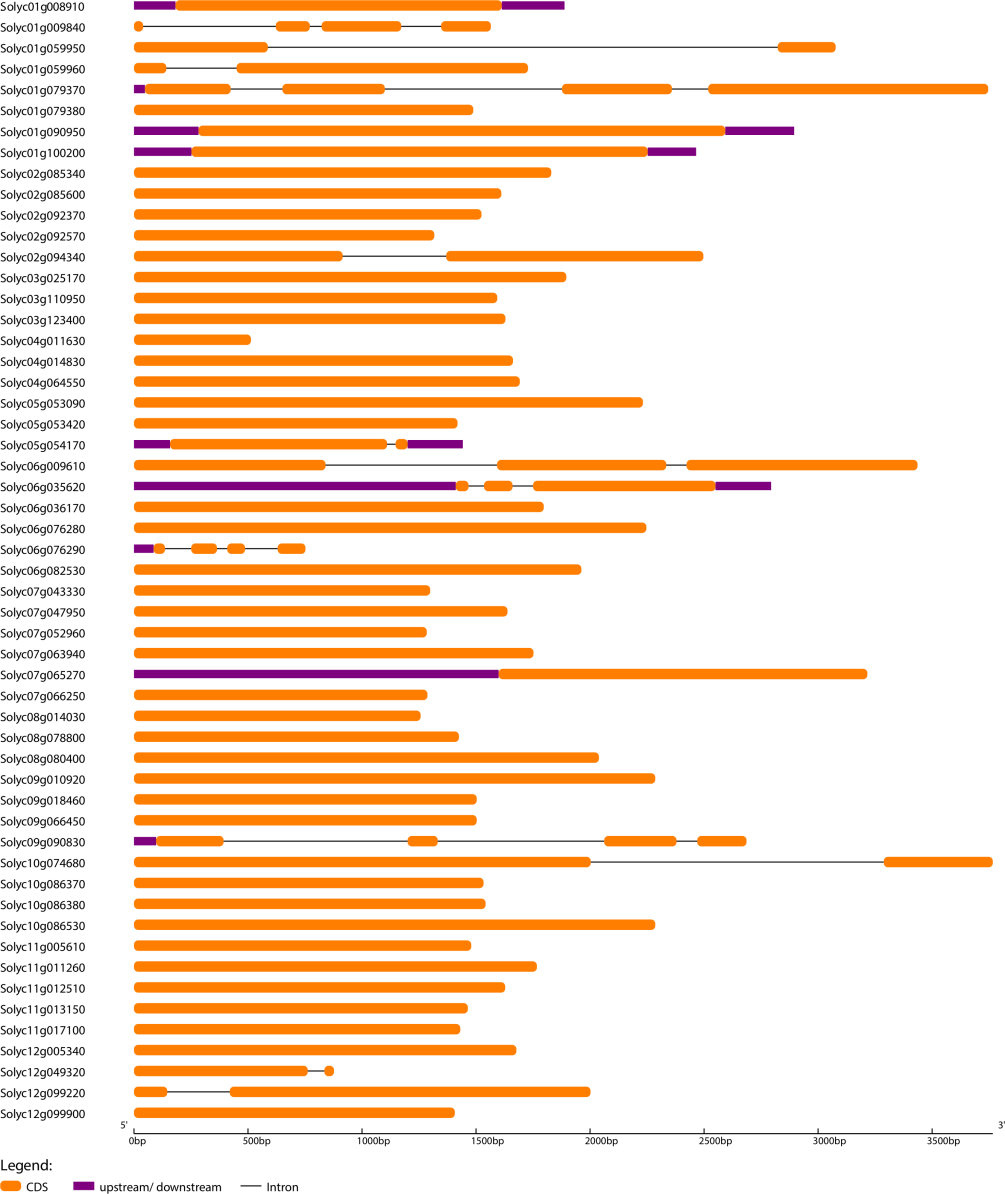
The exon-intron arrangement of *SlWRKY* genes. The arrangement was executed using Gene Structure Display Server 2.0. The exons and introns were represented by green boxes and black lines.

To further understand the evolutionary patterns and gene duplication events, structural and compositional analysis of genes can be used as supporting evidence (Khan et al., 2021). Gene Structure Display Server software was used to generate exon-intron organization maps for *SlWRKY*, *SlGRAS*, and *SlERF* genes (**Figures 3**; **S4**, and **S5**). The outcomes revealed that 38 (70%) of *SlGRAS* and 94 (69%) of *SlERF* and only 2 (2.5%) *SlWRKY* genes have no introns. This evidence suggests that *SlGRAS* and *SlERF* genes may have evolved recently as the evolution of plant species depends heavily on introns, and newly evolved species may have fewer introns than their ancestors [64]. It is worth noting that all members of *SlGRAS* and *SlERF* families possess sample structural organization, showing that there are exons loss or gain during the evolution process of S. lycopersicum genome.

To investigate the compositional diversification among members of the *SlWRKY*, *SlGRAS* and *SlERF* genes, a total of 10 distinct conserved motifs within the proteins were identified using MEME software (**Figures S6**–**S8**). The red-colored motifs were uniformly found in almost all the *SlWRKY*, *SlGRAS*, and *SlERF* proteins, so these motifs significantly represent the conserved *WRKY*, *GRAS*, and *ERF* domains, respectively. The potentially conserved motif (red-colored) was not identified in a few members, such as Solyc03g095770 (*WRKY*), Solyc06g076290 (*GRAS*), and Solyc06g053240 (*ERF*), possibly due to lack of homology, rearrangements or disruption of alignment (Gribskov, 2019). On the other hand, several *WRKY* proteins contained only one or two motifs, which might be attributed to the short duration, resulting in a shorter domain. With a few exceptions, most of the genes in the same phylogenetic clade had the same exon-intron structure and motif composition, indicating that gene structures may have influenced the evolution of these genes and possibly have the same functional roles. For instance, 80% of the *WRKY* members in the clade-I had 3 exons, and 4-7 conserved motifs, and 95% of the *ERF* members in clade-I of the phylogenetic tree are intron-less and most of them have 3 conserved motifs.

### Gene ontology enrichment analysis

**Figure 4:**
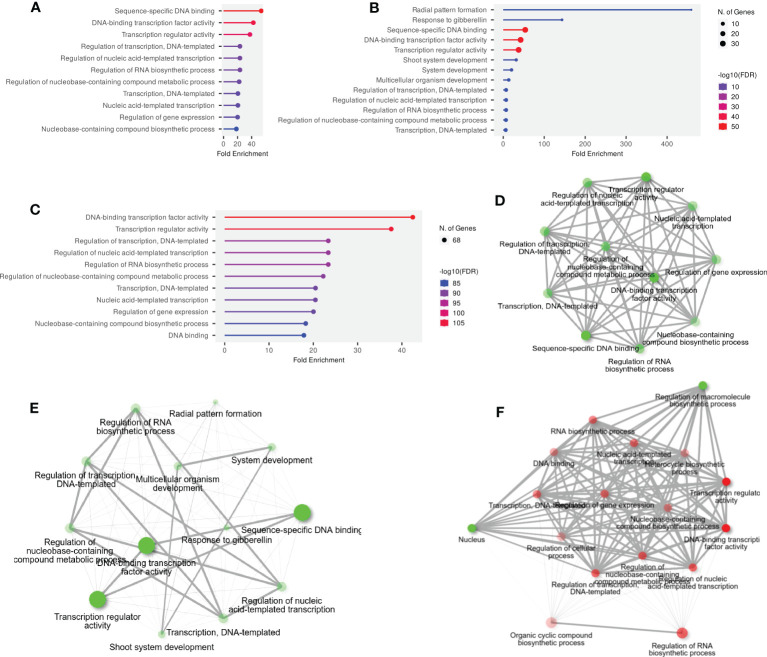
**(A)** The GO annotation of *SlWRKY* genes, **(B)** The GO annotation outcomes of *SlGRAS* genes, **(C)** The GO annotation outcomes of *SlERF* genes, **(D)** GO and KEGG enrichment analyses of *SlWRKY* genes and their expression patterns, **(E)** GO and KEGG enrichment analyses of *SlGRAS* genes and their expression patterns and **(F)** GO and KEGG enrichment analyses of *SlERF* genes and their expression patterns.

GO and KEGG enrichment analyses were performed to advance our understanding of the dynamic roles of *SlWRKY*, *SlGRAS*, and *SlERF* genes at the molecular level. The annotated genes were categorized into the three main functional GO categories: molecular function (MF), biological processes (BP), and cellular component (CC). Out of 51 annotated *SlWRKY* genes, 37 (72.5%), 7 (13.7%), and 5 (9.8%) of the genes were assigned to BP, MF, and CC, respectively (**Figure S9**). GO analysis showed that among the 49 annotated genes, 8 (16.3%), 35 (71.4%), and 6 (12.2%) *GRAS* genes were related to MF, BP, and CC, respectively (**Figure S10**). In the ERF family, the BP category was the highly enriched term with 63 (80.7%), followed by MF with 7 (8.9%) and CC with 6 (7.6%) (**Figure S11**). The GO annotation outcomes of all the genes of the three families presented quite a few substantially enriched terms. For instance, in the BP category, the highly enriched terms were transcription and DNA templated (GO:0006351), regulation of RNA biosynthetic process (GO:2001141), regulation of nucleobase-containing compound biosynthetic process (GO:0019219) and response to gibberellin (GO:0009739). In the MF class, the most enriched terms were sequence-specific DNA binding (GO:0043565), DNA-binding transcription factor activity (GO:0003700), and transcription regulator activity (GO:0140110). Whereas in the CC class, the highly enriched terms were intracellular organelles (GO:0043229) and intracellular anatomical structure (GO:0005622) (**Figures 4A–C**).

The KEGG pathway enrichment study discovered that the highly enriched pathways include MAPK signaling (KEGG:04016), plant-pathogen interaction (KEGG:04626), and hormone signal transduction (KEGG:04075) (**Figures 4D–F**). Moreover, only 2 genes from the WRKY and ERF families were found to be involved in KEGG pathways, but no GRAS genes were connected to these pathways. Briefly, it can be concluded that GO and KEGG pathway enrichment study validates the functional contribution of *SlWRKY, SlGRAS* and *SlERF* genes in several biological, molecular, and cellular processes associated with regulation of gene expression, plant-pathogen interaction, responses to stresses and biosynthesis of different metabolites.

### Functional annotations based on protein-protein interactions

**Figure 5:**
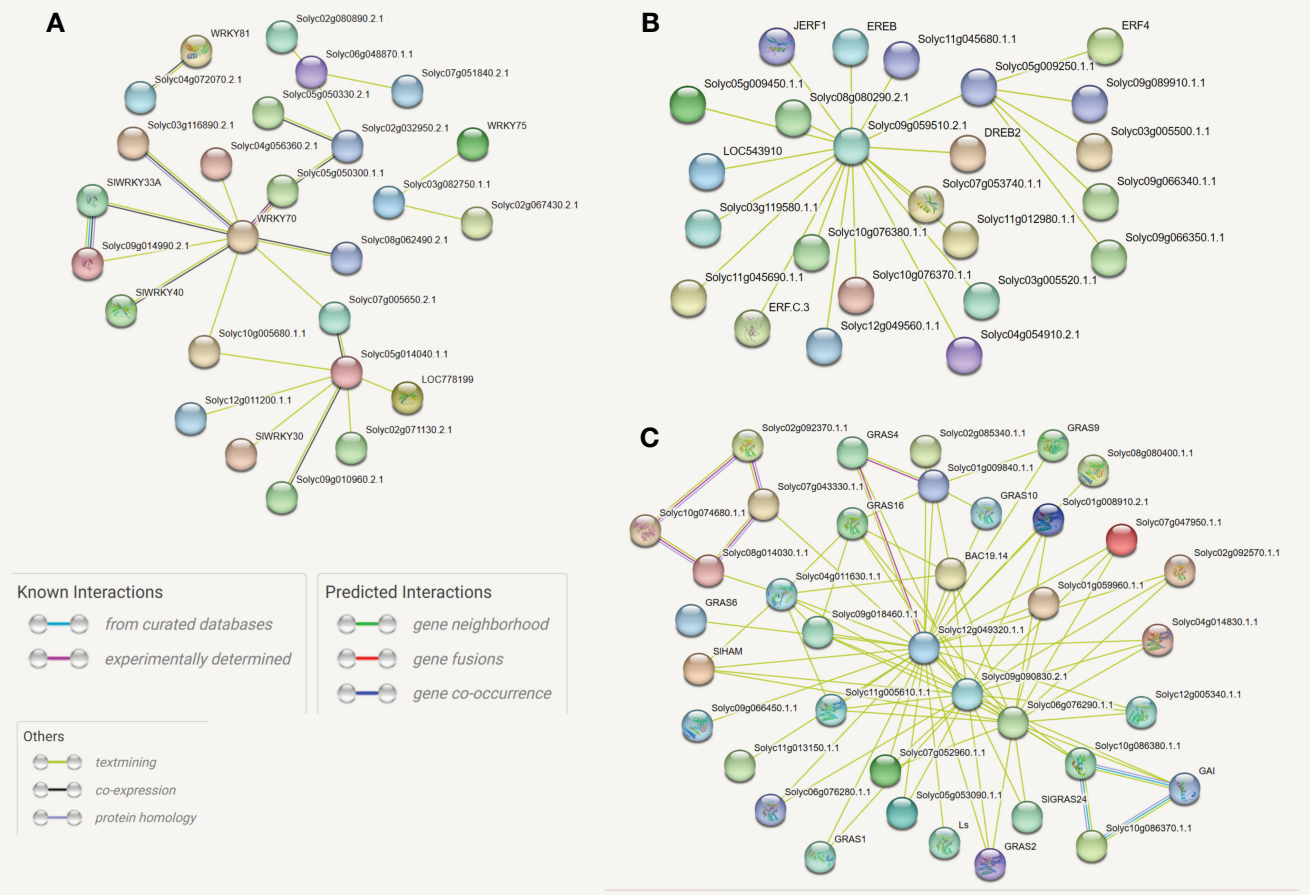
**(A)** Protein-protein association network of the *SlWRKY* genes based on their available information. The online tool STRING was used to predict the entire network. Different line colors represent the type of evidence for the associations, which are shown in the legend. **(B)** Protein-protein association network of the *SlGRAS* genes based on their available information. The online tool STRING was used to predict the entire network. Different line colors represent the type of evidence for the associations, which are shown in the legend. **(C)** Protein-protein association network of the *SlERF* genes based on their available information. The online tool STRING was used to predict the entire network. Different line colors represent the type of evidence for the associations, which are shown in legend.

Protein-protein interactions play crucial roles in the gene expression-regulating process. Interacting proteins often participate in the same biological process and thus are likely to share some function annotations (Peng et al., 2017). The STRING, an online database, was employed to compute the protein-protein interaction network of differentially expressed genes. The outcomes revealed that *WRKY70* (Solyc03g095770), three members of the *GRAS* family (Solyc12g049320, Solyc09g090830, and Solyc10g086380), and one *ERF* member (Solyc09g059510) are the hub proteins in the whole networks. Most *SlGRAS* proteins showed co-expression; however, only 26 and 24 of the *SlWKY* and *SlERF* proteins are linked directly. The constructed phylogenetic trees also supported most of these interactions. Thus, this approach could be used to predict the function of the uncharacterized co-expressed protein. For instance, *SlWRKY70* (Solyc05g014040) is involved in defense against aphids, *Macrosiphum euphorbiae*, and root-knock nematode (Atamian et al., 2012) and has a strong co-expression link with un-characterized Solyc05g050300 (*SlWRKY60*) and Solyc08g062490 (*SlWRKY50*). Similarly, The *GRAS* gene, Solyc02g092370, is involved in meristem formation and trichome differentiation (D'Esposito et al., 2021). However, the specific functions of its strong interactive partners (Solyc10g074680, Solyc07g043330, and Solyc08g014030) are still not comprehensively studied (**Figures 5A–C**).

### Differential expression profiles of *SlWRKY*, *SlGRAS*, and *SlERF* genes during fungal symbiotic association with tomato plants

**Figure 6:**
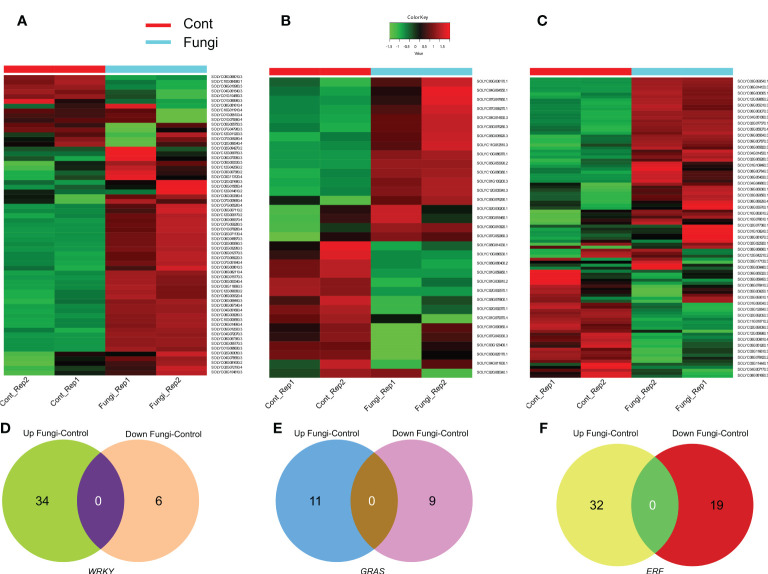
**(A)** Heatmap of differentially expressed *SlWRKY* genes during the symbiotic association of *C lunata* and *S. lycopersicum*. **(B)** Heatmap of differentially expressed *SlGRAS* genes during the symbiotic association of *C lunata* and *S. lycopersicum*. **(C)** Heatmap of differentially expressed *SlERF* genes during the symbiotic association of *C lunata* and *S. lycopersicum*. The dark red, dark green and light green color boxes indicate upregulation, downregulation, and no differential expression, respectively. **(D)** Venn diagram of the differentially expressed *SlWRKY* genes during tomato–fungus interaction **(E)** Venn diagram of the differentially expressed *SlGRAS* genes during tomato–fungus interaction and **(F)** Venn diagram of the differentially expressed *SlERF* genes during tomato–fungus interaction.

Since gene expression patterns are associated with their specific physiological functions; therefore, RNA-seq data was used to evaluate the expression level and elucidate the functional roles of the *SlWRKY*, *SlGRAS*, and *SlERF* genes during the symbiotic association of *C. lunata* and *S. lycopersicum* (**Figures 6A–C**). The results revealed that more than 34 (53%), 11 (33%), and 32 (24%) of the investigated *SlWRKY*, *SlGRAS*, and *SlERF* genes, respectively, significantly exhibited upregulation. For instance, in the *SlWRKY* family, Solyc02g094270 (*SlWRKY38*), Solyc08g067340 (*SlWRKY46*), Solyc06g048870 (*SlWRKY19*) and Solyc04g051690 (*SlWRKY51*), in the *SlGRAS* family, Solyc10g086370 (*SlGLD2*), and Solyc10g086380 (*SlGLD1*) while in *SlERF* family, Solyc02g077370 (*SlERF.C.5*), Solyc06g054630 (*ERF16*) and Solyc09g066350 (*SlERF.B12*). In contrast, about 6 (9%) *SlWRKYs* such as Solyc10g084380 (*SlWRKY44*) and Solyc09g010960 (*SlWRKY49*), 9 (27%) *SlGRASs* such as Solyc08g080400 and Solyc01g059950 and 19 (14%) *SlERF* genes such as Solyc02g092050, Solyc08g078170 and Solyc06g009810 were downregulated significantly during the *C. lunata* and *S. lycopersicum* symbiotic association (**Figures 6D–F**). These results implied that a number of *SlWRKY*, *SlGRAS*, and *SlERF* genes ight help regulate the symbiotic association between the plants and *C. lunata*.

### Possible roles of *SlWRKY*, *SlGRAS*, and *SlERF* genes in hormonal regulation

We investigated differentially expressed transcripts using RNA-seq data and found numerous upregulated candidate transcripts that are likely to be involved in plant hormones’ signal transduction pathways during the development of the symbiotic association of *C. lunata* with *S. lycopersicum*. For instance, *SlWRKY35*, *SlWRKY45*, *SlWRKY39*, *SlWRKY46*, *SlGRAS24*, *SlGRAS4*, *SlGRAS40*, *SlERF.B4*, *SlERF.C3* and *SlERF.A3* (**Figures 6A–C**). Previous studies on these genes provide evidence for the validity of our findings (Sun et al., 2015; Ouyang et al., 2016; Chinnapandi et al., 2017; Huang et al., 2017; Liu et al., 2017; Chinnapandi et al., 2019; Liu et al., 2021; Shu et al., 2021; Asaf et al., 2022; Chen et al., 2022).

### Validation of *SlWRKY, SlGRAS* and *SlERF* genes by RT-qPCR analyses

**Figure 7:**
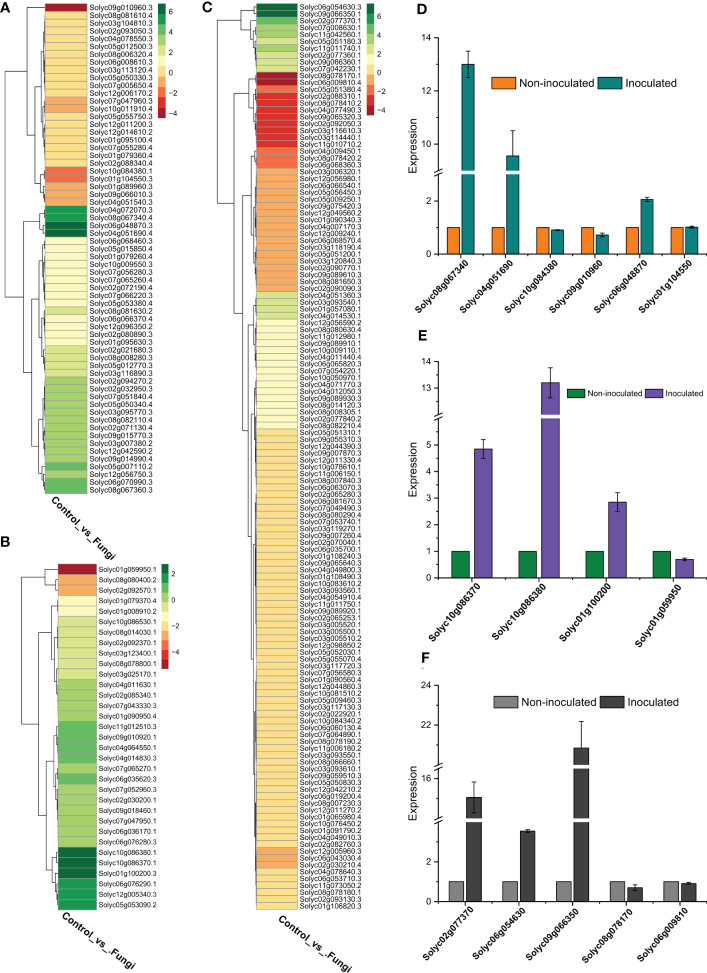
**(A)** Expression profiling of *SlWRKY* genes during the symbiotic association of *C lunata* and *S. lycopersicum*, **(B)** Expression profiling of *SlGRAS* genes during the symbiotic association of *C lunata* and *S. lycopersicum*, **(C)** Expression profiling of *SlERF* genes during the symbiotic association of *C lunata* and *S. lycopersicum*, **(D)** Statistics of DEGs (Upregulated and downregulated) *SlWRKY* genes both in inoculated and non-inoculated tomato plants, **(E)** Statistics of DEGs *SlGRAS* genes both in inoculated and non-inoculated tomato plants, **(F)** Statistics of DEGs *SlERF* genes both in inoculated and non-inoculated tomato plants.

To validate the RNA-seq data accuracy, the relative expression levels of five *SlWRKYs* (Solyc08g067340, Solyc04g051690, Solyc10g084380, Solyc09g010960, Solyc01g104550) four *SlGRASs* (Solyc10g086370, Solyc10g086380, Solyc01g100200, Solyc01g059950) and five *SlERFs* (Solyc02g077370, Solyc06g054630, Solyc09g066350, Solyc08g078170, Solyc06g009810) genes were determined using qRT-PCR with specific primers (**Table S2**). The expression patterns showed a similar trend as shown by RNA-seq data, and a significant association was found between RNA-seq and RT-qPCR results, showing the accuracy of the RNA-seq data. For instance, the expression of Solyc08g067340 and Solyc04g051690 genes of *WRKY* family increased up to 13 and 9-fold respectively as compared to their counterparts in non-inoculated plants. Similar, more than 13 and 2-folds increase, was observed in the expression level of Solyc10g086380 (*SlGRAS*) and Solyc09g066350 (*SlERF*), respectively, during symbiotic association of *C. lunata* and *S. lycopersicum* compared to their counterparts in non-inoculated plants (**Figure 7**).

## Discussion

Endophytic fungi develop a mutualistic symbiotic association with plants, thereby exhibiting a unique potential to synergize plant growth through various mechanisms (Clay, 1988; Wani et al., 2015; Asaf et al., 2022). This study provides a comprehensive study of the potential regulatory roles of *SlWRKY*, *SlGRAS*, and *SlERF* genes in the mutualistic symbiotic association of *S. lycopersicum* and the endophytic fungus *C. lunata* SL1. Through in silico genome-wide analyses, we identified a total of 81 *SlWRKY*, 54 *SlGRAS*, and 137 *SlERF* genes in the *S. lycopersicum* genome. Although most of these genes have yet to be functionally characterized, our study sheds light on their possible roles in endophytic fungal symbiosis. To better understand the regulatory mechanisms involved in this symbiotic association, we analyzed various aspects of the identified genes, including gene duplications, conserved motifs, gene structure, protein-protein interactions, gene ontology, and expression patterns. Through these analyses, we were able to infer important insights into the functional repertoire of the *SlWRKY*, *SlGRAS*, and *SlERF* families, using *Arabidopsis* as a reference for comparison. Combining phylogenetic trees of *S. lycopersicum* WRKY, GRAS, and ERF genes with their Arabidopsis counterparts was constructed to evaluate their evolutionary and functional insights because the higher the sequence similarities, the higher the sequence similarities, the functions of proteins are likely to be more similar in different species (Cantarel et al., 2006; Niu et al., 2017). Our comparative phylogenetic tree analysis revealed that *WRKY*, *GRAS*, and *ERF* genes of *Arabidopsis* and *S. lycopersicum* were present in virtually all clades of the constructed trees and classified into 9, 7, and 11 main clades, respectively, based on tree topologies and sequence similarities. Analysis of the paralogous relationship revealed that members of the *SlERF* family were closely related in comparison to *SlWRKY* and *SlERF* members. A relatively higher number of orthologous gene pairs supported by a high degree of homology were observed in the *Arabidopsis*-*S. lycopersicum WRKY* (27%) showed the existence of ancestral relationships between *Arabidopsis* and *S. lycopersicum WRKY* genes before the divergence of the species as reported previously (Khan et al., 2023).

Analysis of the architecture of conserved protein motifs and gene structure also supported the phylogenetic classification’s reliability. With a few exceptions, a conserved motif analysis revealed that all the *SlWRKY*, *SlGRAS*, and *SlERF* members harbored the typical domains, and each subfamily exhibited similar motif compositions. Remarkably, most of the genes in the same subfamily generally showed similar exon-intron structure, and the intron position and number are almost completely conserved within most subfamilies. It was observed that 70% and 69% of the members of the *GRAS* and *ERF* members have no introns, respectively, and possess sample structural organization. It was discovered that 70% and 69% of *GRAS* and *ERF* members lack introns and possess sample structural organization. This has been reported previously that after segmental duplication, the rate of intron loss is higher than the rate of intron gain (Nuruzzaman et al., 2010). Thus, it can be concluded that most of the *GRAS* and *ERF* genes might be derived by gene duplication with subsequent intron loss as they have no or fewer introns.

The gene duplication events significantly contribute to creating genetic novelty and acquiring of new gene functions in organisms (Magadum et al., 2013). In the current study, approximately 20 and 13 tandemly and segmentally duplicated pairs were found in *SlWRKY* and *SlERF* genes, respectively. The fewer duplicated pairing of *GRAS* genes indicated that they had experienced different evolutionary dynamics and preferential expression. The co-expression network analysis revealed that un-characterized genes, Solyc03g116890 (*SlWRKY39*), Solyc05g050300 (*SlWRKY60*), and Solyc08g062490 (*SlWRKY50*) have a strong association with *SlWRKY70* (Solyc05g014040) which is involved in defense against aphid, *Macrosiphum euphorbiae* and root-knock nematode (Atamian et al., 2012). The *GRAS* gene, Solyc02g092370, is involved in meristem formation and trichome differentiation and forms a strong co-expression network with Solyc10g074680, Solyc07g043330, and Solyc08g014030 which reflect their close regulatory functions. Solyc09g059510 gene of the *ERF* family from the co-expression network with many other un-characterized *ERF* genes such as Solyc08g080290, Solyc11g045680, Solyc05g009450, Solyc05g009450 and Solyc05g009450 etc., while homolog of Solyc09g059510 in *Arabidopsis* (At1g12980) confers cytokinin independent shoot formation and controls cotyledon development (Ikeda et al., 2006).

GO analysis revealed that most of the *SlWRKY*, *SlGRAS* and *SlERF* genes are involved in biological processes and are responsible for transcription regulation, RNA biosynthetic process, and response to gibberellin, homeostasis, and cell division etc. Additionally, the KEGG pathway enrichment analysis indicated that the *SlWRKY*, *SlGRAS*, and *SlERF* genes were largely associated with MAPK signaling, plant-pathogen interaction, and hormone signal transduction.

Previous research has shown that the endophytic fungus *C. lunata* can establish a mutualistic relationship with *S. lycopersicum*, promoting plant growth by producing hormones such as indole acetic acid (IAA) and gibberellins (GAs). Furthermore, during this symbiotic association, the biosynthesis pathways of hormones such as abscisic acid (ABA), jasmonic acid (JA), and salicylic acid (SA) are upregulated in *S. lycopersicum* (Asaf et al., 2022). Given the significant expression of *SlWRKY*, *SlGRAS*, and *SlERF* genes, we investigated their potential regulatory roles in the different hormonal biosynthetic and signaling pathways involved in the development of the *C. lunata* and *S. lycopersicum* symbiotic association. For instance, *SlWRKY35* (Solyc02g021680), which is also highly expressed in the current study, works as a positive regulator for resistance against *Meloidogyne javanica* infection by mediating indole-3-butyric acid and salicylic acid and production pathways (Chinnapandi et al., 2019). *SlWRKY70* (Solyc05g014040) transcript levels are upregulated by salicylic acid (SA) to contribute in basal immunity in tomato (Atamian et al., 2012). However, in the current study, the *SlWRKY70* is not differentially expressed *SlWRKY45* (Solyc08g067360) is significantly upregulated in the current study, which has also been previously reported to suppress the expression of jasmonic acid and salicylic acid marker genes, proteinase inhibitor (PI), and pathogenesis-related protein (PR1) to enhance susceptibility to the root knot nematode; *Meloidogyne javanica* infection (Chinnapandi et al., 2017). In the current study, *SlWRKY39* (Solyc03g116890) was expressed significantly during the symbiotic association of *C. lunata* with *S. lycopersicum*. However, previously, it has been reported that it participated in the SA-mediated signaling pathway, and its overexpression leads to enhanced resistance to multiple stress factors (Sun et al., 2015). *SlWRKY46* (Solyc08g067340) is a highly expressed gene in the current study, which was previously reported to modulate ROS homeostasis and the SA and JA signaling pathways in tomato plants resulting in enhanced susceptibility to *Botrytis cinerea* infection (Shu et al., 2021). *SlGRAS24* (Solyc01g090950) is downregulated in the current study, and it is previously reported to be involved in modulating auxin and gibberellin signaling and participating in various agronomic traits such as plant height, flowering time, root length etc. (Huang et al., 2017). *SlGRAS4* (Solyc01g100200) is significantly upregulated in the current study, suggesting its role in the development of the symbiotic association, while previously, this gene has been reported to play an important role in fruit repining by regulating the expression of ethylene biosynthesis genes. Overexpression of *SlGRAS40* (Solyc08g078800) influences auxin and gibberellin signaling to enhance resistance to abiotic stresses (Liu et al., 2017). *Botrytis cinerea* infection induced the expression of *SlERF.B4 (*Solyc03g093540), *SlERF.C3* (Solyc09g066360), and *SlERF.A3* (Solyc05g052050) by modification of signaling pathways of stress-related hormones such as salicylic acid, methyl jasmonate and ethylene (Ouyang et al., 2016). Our study is consistent with previous reports, as we also observed upregulation of the aforementioned genes during the development of the symbiotic association between *S. lycopersicum* and the endophytic fungus C*. lunata*, indicating their potential involvement in this process. It has been reported that expression of *SlERF.F5* (Solyc10g009110) negatively affects plant growth, fruit repining, and leaf senescence by affecting the expression levels of genes in the ethylene and jasmonic acid biosynthesis and signal transduction pathways (Chen et al., 2022). However, in the current study up-regulation of *SlERF.F5* suggests its involvement in development of the symbiotic association of *C. lunata* with *S. lycopersicum*.

Our RNA-seq and qRT-PCR analysis revealed that most of the *SlWRKY*, *SlGRAS*, and *SlERF* genes significantly exhibited upregulation expression levels during the symbiotic association of *C. lunata* and *S. lycopersicum*. For instance, in *SlWRKY* family, Solyc02g094270 (*SlWRKY38*), Solyc08g067340 (*SlWRKY46*), Solyc06g048870 (*SlWRKY19*), Solyc08g067360 (*SlWRKY45*), Solyc04g051690 (*SlWRKY51*), Solyc04g072070 (*SlWRKY55*) and Solyc05g007110 (*SlWRKY76*) were highly expressed while Solyc10g084380 (*SlWRKY44*), Solyc09g010960 (*SlWRKY49*), Solyc09g066010 (*SlWRKY24*) and Solyc01g104550 (*SlWRKY9*) showed downregulation. In *SlERF* family, the highly expressed genes include Solyc02g077370 (*SlERF.C.5*), Solyc02g077360 (*SlERF.B.2*), Solyc09g066360 (*SlERF.C3*), Solyc07g008630 (*LeEIX2*), Solyc07g042230 (*SlERF.H9*), Solyc11g042560 (ERF4), Solyc06g054630 (*ERF16*), Solyc09g066350 (*SlERF.B12*) and Solyc11g011740 (*ERF2*). In contrast, Solyc11g010710, Solyc05g051380, Solyc04g077490 (*ANT*), Solyc02g092050, Solyc09g065320, Solyc04g011440, Solyc02g088310, Solyc08g078170 (*SlERF.A4*), Solyc06g009810 and Solyc03g116610 (*SlSHN1*) were found to be highly downregulated. In the *GRAS* family, Solyc10g086370 (*SlGLD2*), Solyc10g086380 (*SlGLD1*), and Solyc01g100200 (*SlGRAS4*) were significantly expressed, while the majority of the *GRAS* genes were upregulated during the symbiotic association of *C. lunata* and *S. lycopersicum*.

Future studies can focus on elucidating the regulatory mechanisms that trigger and control the expression of fungal and plant signaling genes and the developmental pathways leading to endophytic symbiosis. This can be done by employing transcriptomics, proteomics, and metabolomics approaches to study the changes in gene expression, protein abundance, and metabolite profiles during the establishment and maintenance of endophytic symbiosis. Furthermore, advanced techniques such as single-cell RNA sequencing and CRISPR-Cas9 genome editing can be employed to gain deeper insights into the regulatory mechanisms underlying this symbiotic association.

## Conclusion

The current study on the regulatory functions of *WRKY*, *GRAS*, and *ERF* genes in *S. lycopersicum* during symbiosis development with an endophytic fungus *C. lunata* provides a comprehensive understanding of the potential roles of these genes in endophytic fungal symbiosis. The findings of this study have significant implications for future research. For instance, the functional characterization of the identified *SlWRKY*, *SlGRAS*, and *SlERF* genes will provide a more in-depth understanding of their roles in endophytic fungal symbiosis. Furthermore, the co-expression network analysis identified several uncharacterized genes that showed strong associations with known genes, which could be potential candidates for further investigation. Additionally, the GO analysis revealed the involvement of these genes in various biological processes, such as transcription regulation, RNA biosynthetic processes, and response to gibberellin, suggesting their potential applications in agricultural biotechnology for improving plant growth and productivity. Future research can focus on investigating the specific roles of the identified genes in endophytic fungal symbiosis and their interactions with the plant host. Moreover, the functional characterization of these genes in other plant species and their interactions with different endophytic fungi will provide a more comprehensive understanding of their roles in plant-microbe interactions. The results of this study also provide a basis for the development of novel strategies for improving plant growth and productivity through the manipulation of these genes. Thus, this study opens up new avenues for future research in the field of plant-microbe interactions and agricultural biotechnology.

## Data availability statement

The data presented in the study are deposited in the National Center for Biotechnology Information (NCBI) repository, accession number PRJNA913645.

## Author contributions

‘IK’, ‘SA’ and ‘L’ wrote the original draft. SB ‘AK’ and ‘RJ’ collected all the data and drew the phylogenetic tree and revised the original draft. K-MK and AA-H supervised and arranged resources. All authors contributed to the article and approved the submitted version.

## Supplementary material

Supplementary Figure 1 **(A)** The neighbor-joining phylogenetic tree of the 137 *SlERF* proteins constructed with MEGA-11 with 1000 times replicate. The major 7 phylogenetic clades are marked as I to VII, respectively. **(B)** Comparative phylogenetic tree of WRKY genes of Arabidopsis and *S. lycopersicum*. Multiple sequence alignment of full-length WRKY proteins was done using ClustalW, and the phylogenetic tree was constructed using MEGA-11 by the neighbor-joining method with 1000 bootstrap replicates. The tree was divided into 9 phylogenetic clades marked with different colors.

Supplementary Figure 2 Chromosomal mapping of *SlWRKY*, *SlGRAS* and *SlERF* genes on *S. lycopersicum* genome. Chromosomal positions of the genes were mapped on the basis of the tomato genome database SGN. The chromosome number is indicated above each chromosome. *SlWRKY*, *SlGRAS*, and *SlERF* genes were colored green, red, and blue, respectively. The scale is in megabases (Mb).

Supplementary Figure 3 Chromosomal positions and inter-chromosomal groups of duplicated *SlWRKY*, *SlGRAS*, and *SlERF* gene pairs. Green, red, and blue colors indicate *SlWRKY*, *SlGRAS* and *SlERF*, respectively.

Supplementary Figure 4 The exon-intron arrangement of *SlGRAS* genes. The arrangement was executed using Gene Structure Display Server 2.0. Yellow boxes and black lines represented the exons and introns.

Supplementary Figure 5 The exon-intron arrangement of *SlERF* genes. The arrangement was executed using Gene Structure Display Server 2.0. The exons and introns were represented by light blue boxes and black lines.

Supplementary Figure 6 Conserved motifs analysis of *SlWRKY* protein family by MEME program. The red-colored motif was uniformly found in almost all the *SlWRKY* proteins may the conserved WRKY domain.

Supplementary Figure 7 Conserved motifs analysis of *SlGRAS* protein family by MEME program. The red-colored motif was uniformly found in almost all the *SlGRAS* proteins may the conserved GRAS domain.

Supplementary Figure 8 Conserved motifs analysis of *SlERF* protein family by MEME program. The red-colored motif was uniformly found in almost all the *SlERF* proteins may the conserved ERF domain.

Supplementary Figure 9 Gene Ontology and KEGG enrichment analysis of the *SlWRKY* genes.

Supplementary Figure 10 Gene Ontology KEGG enrichment analysis of the *SlGRAS* genes.

Supplementary Figure 11 Gene Ontology KEGG enrichment analysis of the *SlERF* genes.

Supplementary Table 1 The data of 81 *WRKY*, 54 *GRAS*, and 137 *ERF* genes were identified in *S. lycopersicum* genome.

Supplementary Table 2 Designed primers for RT-qPCR analysis of the selected genes.
