# Supplementary figures and images for: Dynamic interplay of WRKY, GRAS, and ERF transcription factor families in tomato-endophytic fungal symbiosis: insights from transcriptome and genome-wide analysis
Source: Front Plant Sci. 2023 Jun 5;14:1181227. doi: 10.3389/fpls.2023.1181227 (PMC10277700; doi:10.3389/fpls.2023.1181227)

**A**

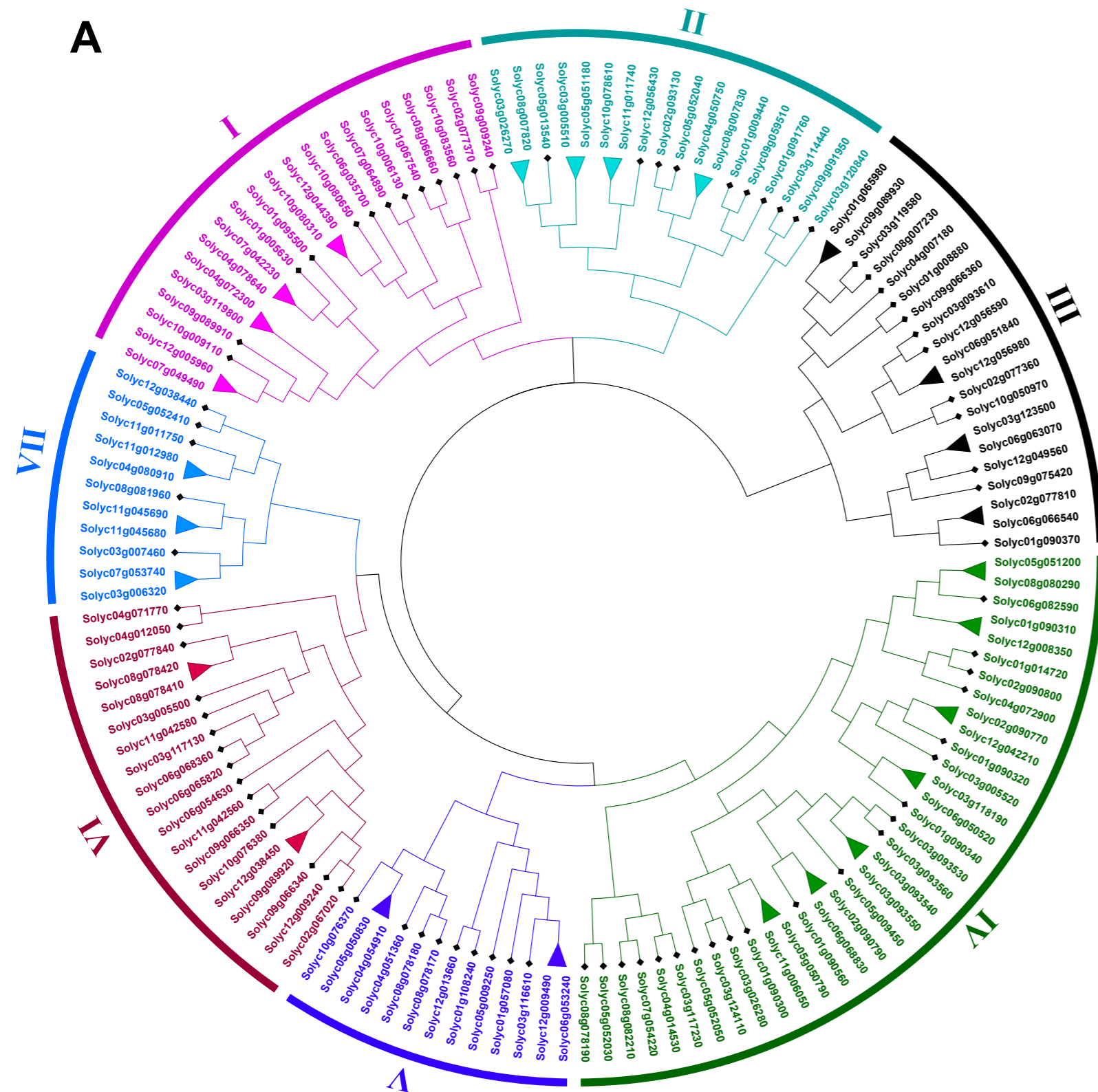

# B

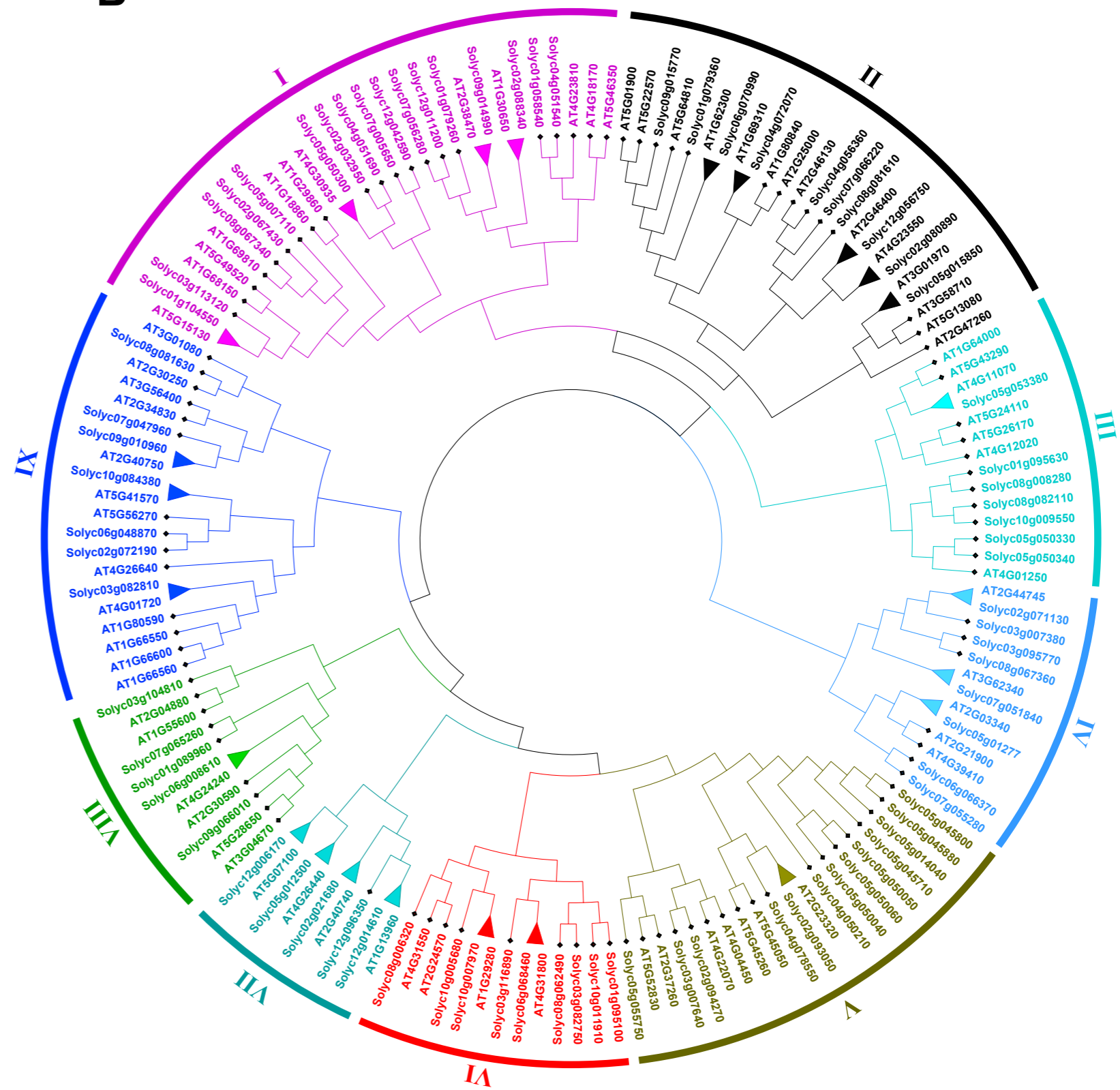

Supplement: Supplementary Figure 1 — (A) The neighbor-joining phylogenetic tree of the 137 SlERF proteins constructed with MEGA-11 with 1000 times replicate. The major 7 phylogenetic clades are marked as I to VII, respectively. (B) Comparative phylogenetic tree of WRKY genes of Arabidopsis and S. lycopersicum. Multiple sequence alignment of full-length WRKY proteins was done using ClustalW, and the phylogenetic tree was constructed using MEGA-11 by the neighbor-joining method with 1000 bootstrap replicates. The tree was divided into 9 phylogenetic clades marked with different colors. [file DataSheet_1.pdf]

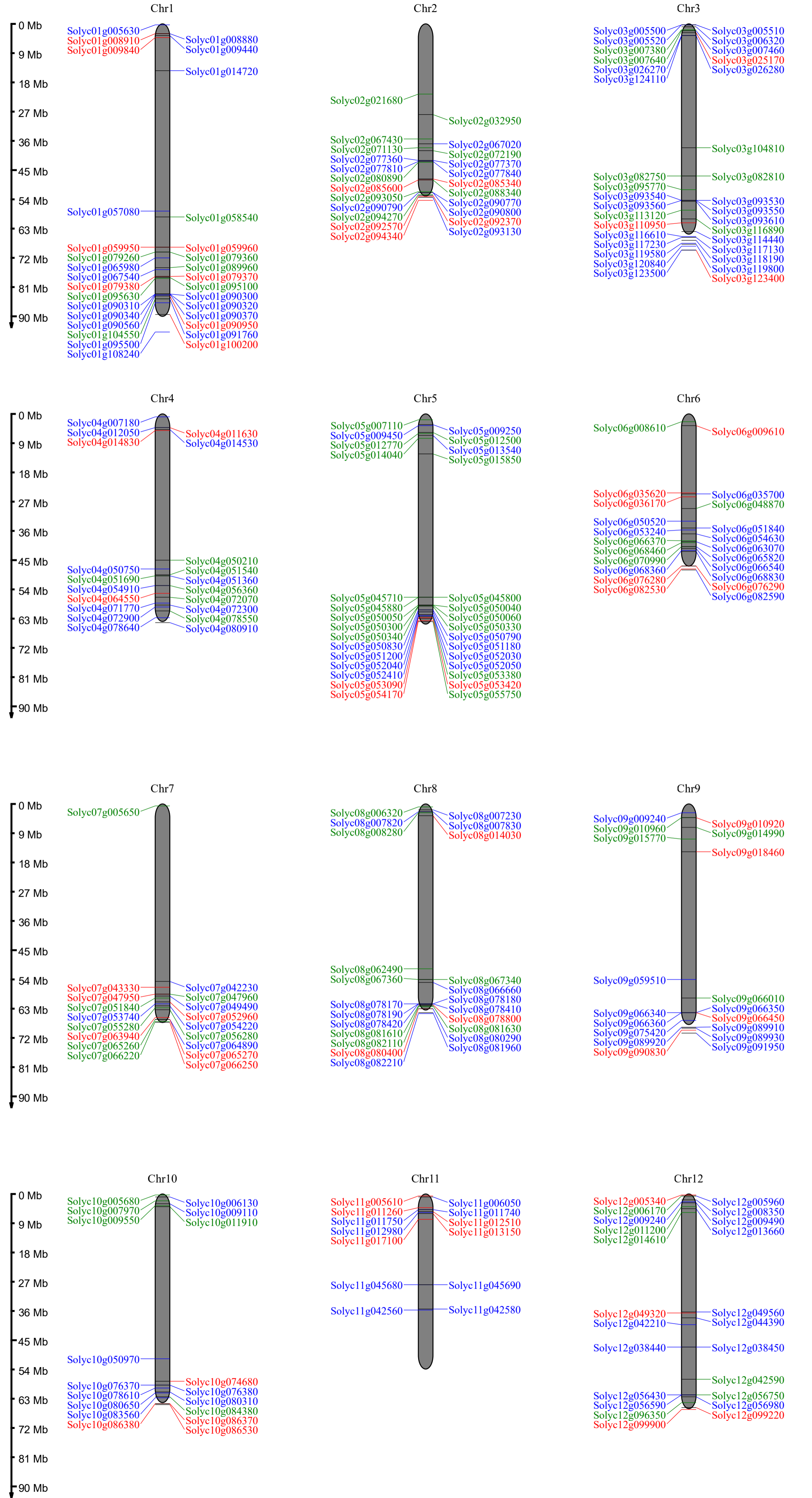

Supplement: Supplementary Figure 2 — Chromosomal mapping of SlWRKY, SlGRAS and SlERF genes on S. lycopersicum genome. Chromosomal positions of the genes were mapped on the basis of the tomato genome database SGN. The chromosome number is indicated above each chromosome. SlWRKY, SlGRAS, and SlERF genes were colored green, red, and blue, respectively. The scale is in megabases (Mb). [file DataSheet_2.pdf]

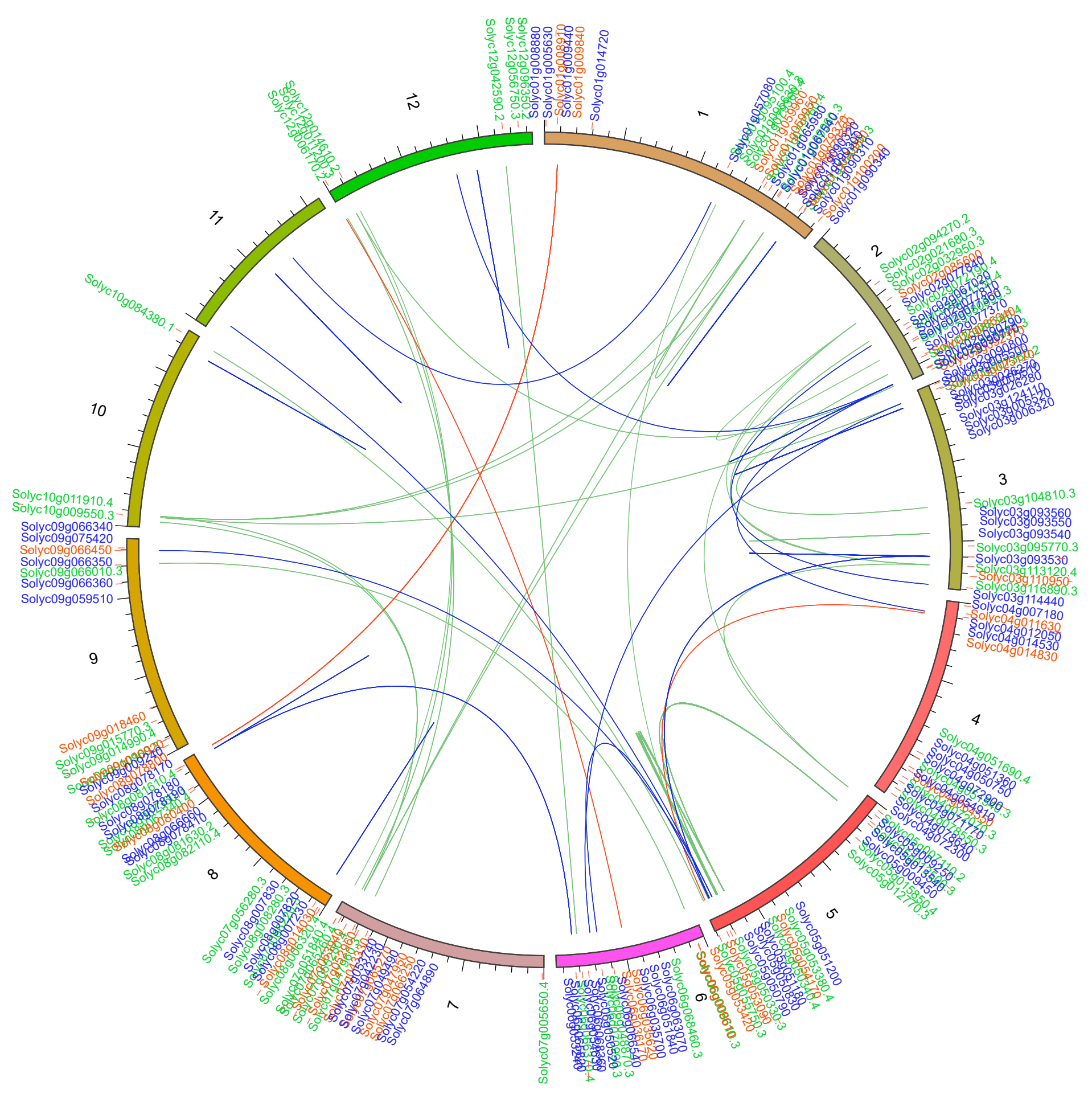

Supplement: Supplementary Figure 3 — Chromosomal positions and inter-chromosomal groups of duplicated SlWRKY, SlGRAS, and SlERF gene pairs. Green, red, and blue colors indicate SlWRKY, SlGRAS and SlERF, respectively. [file DataSheet_3.pdf]

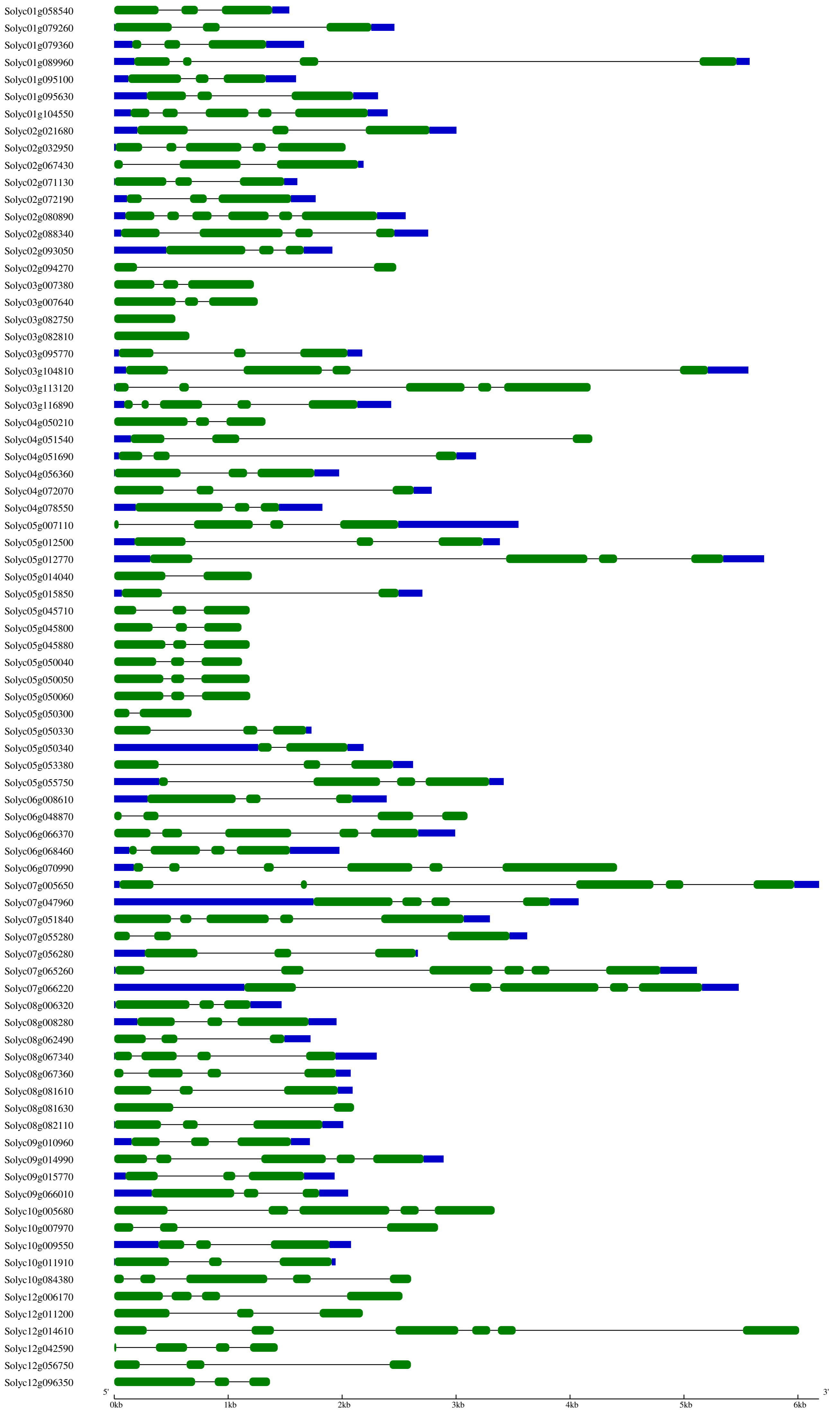

Supplement: Supplementary Figure 4 — The exon-intron arrangement of SlGRAS genes. The arrangement was executed using Gene Structure Display Server 2.0. Yellow boxes and black lines represented the exons and introns. [file DataSheet_4.pdf]

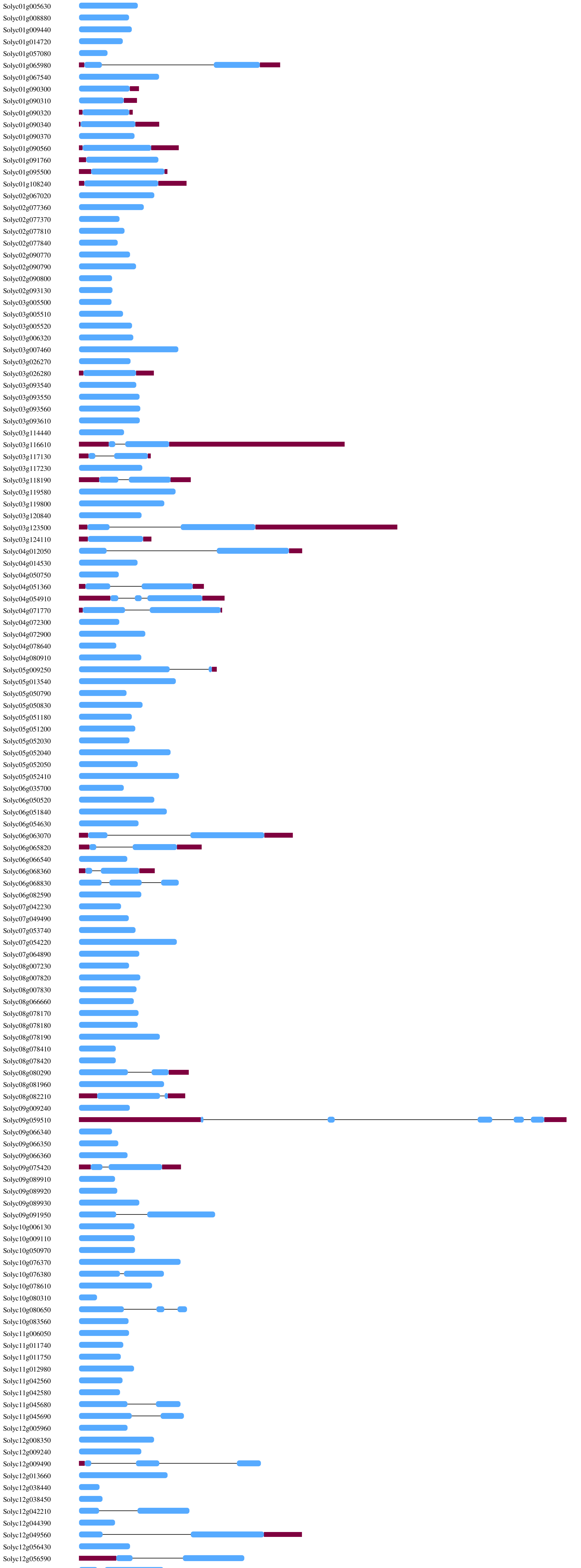

Supplement: Supplementary Figure 5 — The exon-intron arrangement of SlERF genes. The arrangement was executed using Gene Structure Display Server 2.0. The exons and introns were represented by light blue boxes and black lines. [file DataSheet_5.pdf]

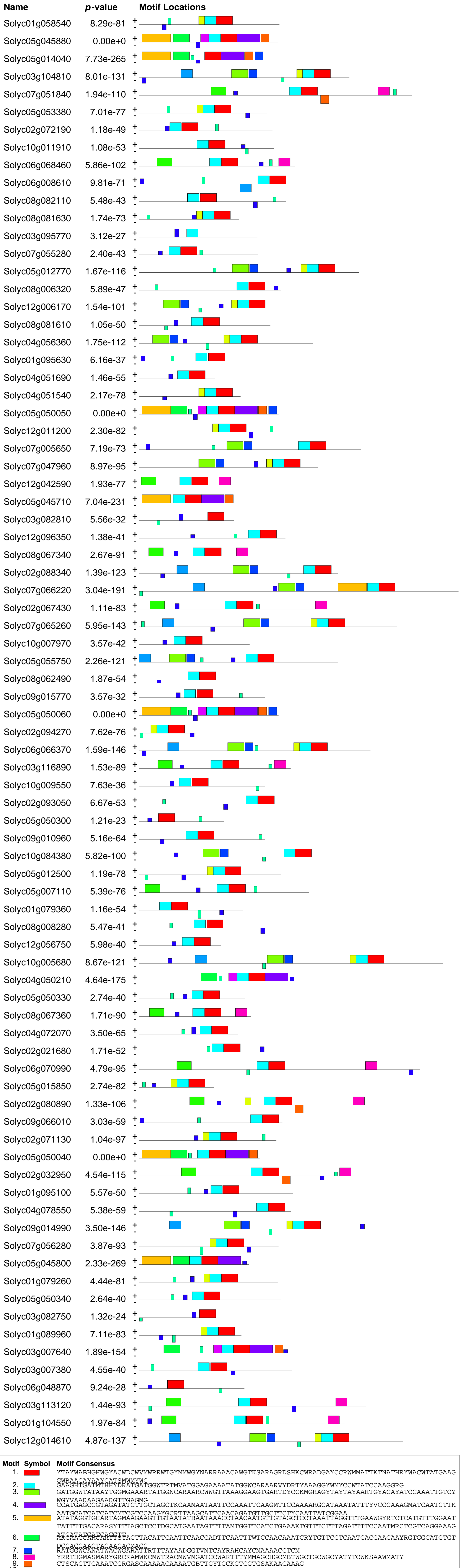

Supplement: Supplementary Figure 6 — Conserved motifs analysis of SlWRKY protein family by MEME program. The red-colored motif was uniformly found in almost all the SlWRKY proteins may the conserved WRKY domain. [file DataSheet_6.pdf]
