# Supplementary material for: Dynamic interplay of WRKY, GRAS, and ERF transcription factor families in tomato-endophytic fungal symbiosis: insights from transcriptome and genome-wide analysis
Source: Front Plant Sci. 2023 Jun 5;14:1181227. doi: 10.3389/fpls.2023.1181227 (PMC10277700; doi:10.3389/fpls.2023.1181227)
Supplement: Supplementary Figure 8 — Conserved motifs analysis of SlERF protein family by MEME program. The red-colored motif was uniformly found in almost all the SlERF proteins may the conserved ERF domain. [file DataSheet_8.pdf]

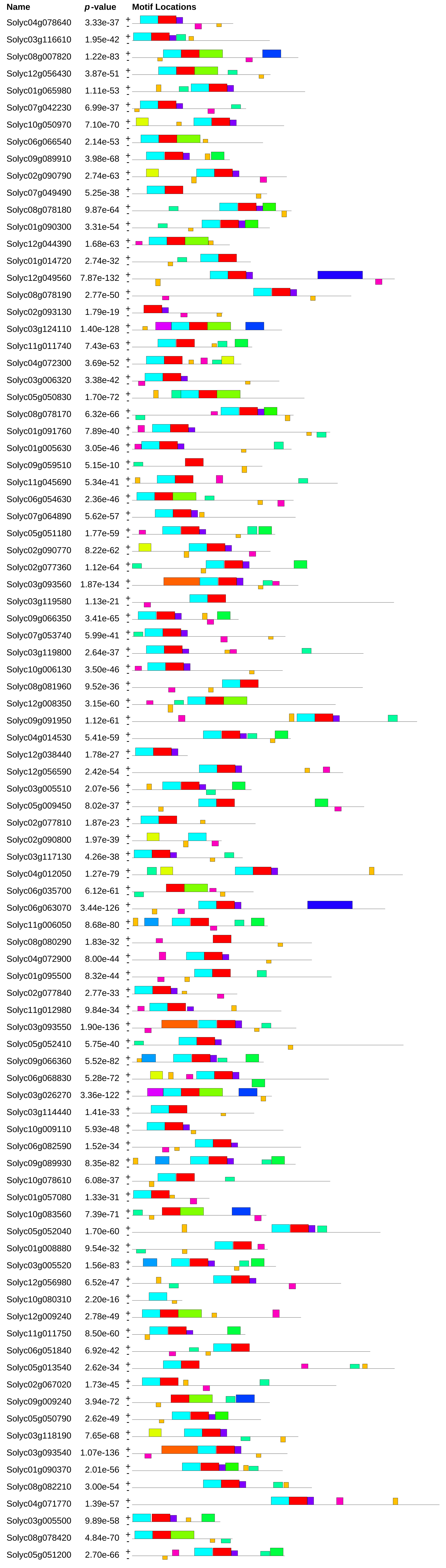

Motif	Symbol	Motif Consensus
1.		CWMGRVTTTGGYTWGGWACWTTTGATACTGCTGAAGADGCTGCTAKRGCTTATGAYRAAGCWGCWWTTRVVHTNMGWGGW
2.		RADVADAARMADTAYAGAGGWGTWMGAMARAGRCCATGGGGWAAATGGGYDGCKGARATWMGAGAYCCDAVDARRAADR
3.		MRYTMAGCTWWWYTNAATTTYCCTGAHTYWGYTBRBYVDTTBCCTVDHCCDGCTWCNWHBTMHCCTARAGAYATWCAARMDGCDGCHGCWMARGCYGCHGMWR
4.		MWAAWGCWADAHTBAATTTYCCWNWDNA
5.		WTCHTCWTCTTCHTCWTCHTC
6.		TGTTNTKGWDTTTGARKATTTKGRTGMTRATTWWTTRGADGAACTTYTDDBTTBTDA
7.		ABTTYRTGGATGAGGANGYGATRTTTKAYATGCCARRWTTWCTTGHDAATATGGCWGAAGGRMTKHTRYTWMCTCCACCWC
8.		CATCAHCAHCANCASCANCATCADCAKCA
9.		TACYTCAAAAACAGAGTAYGATGGTTTTTTCRAATTTGAGGCAAAACSACATGTTATCMRTTCAAATTCYCCGAAACAGAGTAACTTGAGAGAACGGAAGCCATCTCTGAACRTWGCRATACCSGCGAAGCCWRTTGTTGTTGTAGAGAAYGTTGAGA
10.		VTTAVTNVWSAASAWGADWTKTCTDHYATGGTTTCWGCTCTMHMWCATGTTRTY
11.		RATYGRYTCGRRYGWRCCBGAGCCRGYTAGAGTKASGBCKARGMRDCGWTCRMNNTC
12.		WDRATRAWAADAATRADRADRWKAATAAWRATDATVADAAT
13.		MYYTTCSWTTNAAHGWGAAYGAYTCHGAAGABATGGTWHTTYATRRDBTTYTWRMAGAAGC
14.		TTGGGSWGARMCRTGYKCMAGGACTCCAGASATMTCATCTGTTCTGTCRGMWGYTMTDGAAWSYRATGARRCTCAWTTTRWTGAWGRTKYCRRHYYDSAGRARAARYYRAARTCHTGYWCCARCAMYYYVKTRVCWRWTRAYGKDAACACYGWDMMYAHGSYAYCTGAAGWRYTWYCRRCTTTTGAAYCYCAGWTGAA
15.		TGAVGARGWADTTATTTTAGCTTCGAATAAHCCGAARAAGCCAGCTGGCAGRAAGAAGTTTCGAGAAAC
