# Supplementary material for: Dynamic interplay of WRKY, GRAS, and ERF transcription factor families in tomato-endophytic fungal symbiosis: insights from transcriptome and genome-wide analysis
Source: Front Plant Sci. 2023 Jun 5;14:1181227. doi: 10.3389/fpls.2023.1181227 (PMC10277700; doi:10.3389/fpls.2023.1181227)
Supplement: Supplementary Table 2 — Designed primers for RT-qPCR analysis of the selected genes. [file Table_2.docx]

| *SlWRKYs* | Forward | Revers |
| --- | --- | --- |
| Solyc08g067340 | TTTGCACCATCATGTCCAGT | AACGAAGACGCCATTTGTTC |
| Solyc04g051690 | GGAGGTGTTGGATGATGGAT | TGAGGACTCTCGTGGTTGTG |
| Solyc10g084380 | GATCAGCGACGAGAAAAAGC | TGGGACCTCTTCTGTGCTCT |
| Solyc09g010960 | GGGTTGGGAAGAGGGTTAGA | GCTCCACATCGTGGGTTAGT |
| SOLYC06G048870 | GTCCAGCCAAGAAGCAAGTC | CAATAGCAATGGTGCAGGTG |
| SOLYC01G104550 | CAATTCCCCTTTGCAAGTTC | TGAGCGAAGAAATAGCAGCA |
|  |  |  |
| *SlGRASs* |  |  |
| Solyc10g086370 | TCCTCAAATTTGCCCACTTC | GGTTGGCCACTAATTCCTGA |
| Solyc10g086380 | AATTTGGTGGCTTGTGAAGG | GCGACTATGCCAACTCAACA |
| Solyc01g100200 | GTTACAAAGTGGCAGCAGCA | CTTCATTAGGCGGCGAGTAG |
| Solyc01g059950 | CATCGAAACTGAGGCAAACA | CAAAGCCCTCCATACTTCCA |
|  |  |  |
| *SlERFs* |  |  |
| Solyc02g077370 | TGGTTCCAACTCCTCAAAGTG | GCACCATGTCTAGCCGAATC |
| Solyc06g054630 | TCATGATCACGAGGGCAATA | GAGGCCCACTTGCATTGTAT |
| Solyc09g066350 | AACAGCTGAAGAAGCGGCTA | GCCGAAGCAGAAGAAGAAGA |
| Solyc08g078170 | AAAATGGGCAAAGAGTGTGG | ATCCGACGACGATGAAGAAC |
| Solyc06g009810 | GAAGAATCCCAAAACGGTCA | ACGGTGGTAAATCGCAGAAG |

**Designed primers for RT-qPCR analysis of the selected genes.**
