# Supplementary material for: Dynamic interplay of WRKY, GRAS, and ERF transcription factor families in tomato-endophytic fungal symbiosis: insights from transcriptome and genome-wide analysis
Source: Front Plant Sci. 2023 Jun 5;14:1181227. doi: 10.3389/fpls.2023.1181227 (PMC10277700; doi:10.3389/fpls.2023.1181227)
Supplement: Supplementary Figure 7 — Conserved motifs analysis of SlGRAS protein family by MEME program. The red-colored motif was uniformly found in almost all the SlGRAS proteins may the conserved GRAS domain. [file DataSheet_7.pdf]

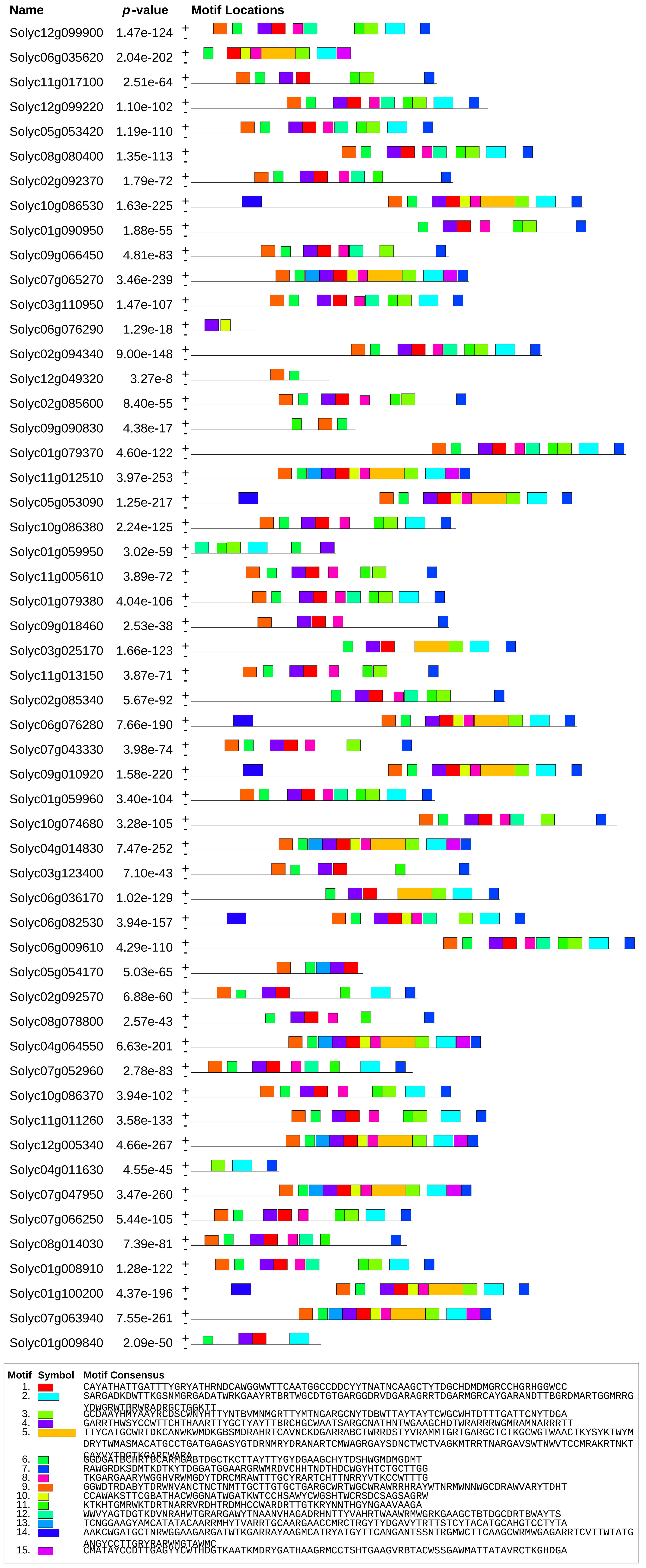
Name
p-value
Motif Locations
Solyc12g099900 1.47e-124
Solyc06g035620 2.04e-202
Solyc11g017100 2.51e-64
Solyc12g099220 1.10e-102
Solyc05g053420 1.19e-110
Solyc08g080400 1.35e-113
Solyc02g092370 1.79e-72
Solyc10g086530 1.63e-225
Solyc01g090950 1.88e-55
Solyc09g066450 4.81e-83
Solyc07g065270 3.46e-239
Solyc03g110950 1.47e-107
Solyc06g076290 1.29e-18
Solyc02g094340 9.00e-148
Solyc12g049320 3.27e-8
Solyc02g085600 8.40e-55
Solyc09g090830 4.38e-17
Solyc01g079370 4.60e-122
Solyc11g012510 3.97e-253
Solyc05g053090 1.25e-217
Solyc10g086380 2.24e-125
Solyc01g059950 3.02e-59
Solyc11g005610 3.89e-72
Solyc01g079380 4.04e-106
Solyc09g018460 2.53e-38
Solyc03g025170 1.66e-123
Solyc11g013150 3.87e-71
Solyc02g085340 5.67e-92
Solyc06g076280 7.66e-190
Solyc07g043330 3.98e-74
Solyc09g010920 1.58e-220
Solyc01g059960 3.40e-104
Solyc10g074680 3.28e-105
Solyc04g014830 7.47e-252
Solyc03g123400 7.10e-43
Solyc06g036170 1.02e-129
Solyc06g082530 3.94e-157
Solyc06g009610 4.29e-110
Solyc05g054170 5.03e-65
Solyc02g092570 6.88e-60
Solyc08g078800 2.57e-43
Solyc04g064550 6.63e-201
Solyc07g052960 2.78e-83
Solyc10g086370 3.94e-102
Solyc11g011260 3.58e-133
Solyc12g005340 4.66e-267
Solyc04g011630 4.55e-45
Solyc07g047950 3.47e-260
Solyc07g066250 5.44e-105
Solyc08g014030 7.39e-81
Solyc01g008910 1.28e-122
Solyc01g100200 4.37e-196
Solyc07g063940 7.55e-261
Solyc01g009840 2.09e-50
Motif Symbol Motif Consensus
1. CAYATHATTGATTTYGRYATHRNDCAWGGWWTTCAATGGCCDDCYYTNATNCAAGCTYTDGCHDMDMGRCCHGRHGGWCC
2. SARGADKDWTTKGSNMGRGADATWRKGAAYRTBRTWGCDTGTGARGGDRVDGARAGRRTDGARMGRCAYGARANDTTBGRDMARTGGMRRG YDWGRWTBRWRADRGCTGGKTT
3. GCDAAYHMYAAYRCDSCWNYHTTYNTBVMNMGRTTYMTNGARGCNYTDBWTTAYTAYTCWGCWHTDTTTGATTCNYTDGA
4. GARRTHWSYCCWTTCHTHAARTTYGCTYAYTTBRCHGCWAATSARGCNATHNTWGAAGCHDTWRARRRWGMRAMNARRRTT
5. TTYCATGCWRTDKCANWKWMDKGBSMDRAHRTCAVNCKDGARRABCTWRRDSTYVRAMMTGRTGARGCTCTKGCWGTWAACTKYSYKTWYM DRYTWMASMACATGCCTGATGAGASYGTDRNMRYDRANARTCMWAGRGAYSDNCTWCTVAGKMTRRTNARGAVSWTNWVTCCMRAKRTNKT CAYVYTDGTKGARCWARA
6. GGDGATBCHRTBCARMGABTDGCTKCTTAYTTYGYDGAAGCHYTDSHWGMDMGDMT
7. RAWGRDKSDMTKDTKYTDGGATGGAARGRWMRDVCHHTNDTHDCWGYHTCTGCTTGG
8. TKGARGAARYWGGHVRWMGDYTDRCMRAWTTTGCYRARTCHTTNRRYVTKCCWTTTG
9. GGWDTRDABYTDRWNVANCTNCTNMTTGCTTGTGCTGARGCWRTWGCWRAWRRHRAYWTNRMWNNWGCDRAWVARYTDHT
10. CCAWAKSTTCGBATHACWGGNATWGATKWTCCHSAWYCWGSHTWCRSDCSAGSAGRW
11. KTKHTGMRWKTDRTNARRVRDHTRDMHCCWARDRTTGTKRYNNTHGYNGAAVAAGA
12. WWVYAGTDGTKDVNRAHWTGRARGAWYTNAANVHAGADRHNTTYVAHRTWAAWRMWGRKGAAGCTBTDGCDRTBWAYTS
13. TCNGGAAGYAMCATATACAARRMHYTVARRTGCAARGAACCMRCTRGYTYDGAVYTRTTSTCYTACATGCAHGTCCTYTA
14. AAKCWGATGCTNRWGGAAGARGATWTKGARRAYAAGMCATRYATGYTTCANGANTSSNTRGMWCTTCAAGCWRMWGAGARRTCVTTWTATG ANGYCCTTGRYRARWMGTAWMC
15. CMATAYCCDTTGAGYYCWTHDGTKAATKMDRYGATHAAGRMCCTSHTGAAGVRBTACWSSGAWMATTATAVRCTKGHDGA
